# Clarithromycin use and the risk of mortality and cardiovascular events: A systematic review and meta-analysis

**DOI:** 10.1371/journal.pone.0226637

**Published:** 2019-12-27

**Authors:** Ching-Hui You, Cheng-Kuan Lin, Po-Hua Chen, Suna Park, Yi-Yun Chen, Nazleen Khan, Stefania I. Papatheodorou, Szu-Ta Chen

**Affiliations:** 1 Department of Epidemiology, Harvard T.H. Chan School of Public Health, Boston, MA, United States of America; 2 Department of Environmental Health, Harvard T.H. Chan School of Public Health, Boston, MA, United States of America; Karolinska Institutet, SWEDEN

## Abstract

**Background:**

Although studies reported increased cardiovascular (CV) risks in patients treated with macrolides, the risks remain controversial among clarithromycin (CLR) users. We aimed to summarize the association between CLR use and the risks of mortality and CV events.

**Methods:**

We searched PubMed, EMBASE, Web of Science, and the Cochrane Library for randomized controlled trials (RCTs) and observational studies with population exposed to CLR published until December 31^st^, 2018. These studies reported either all-cause mortality (primary outcome) or CV adverse events (secondary outcomes) based on multivariate models. Effect measures were synthesized by study design and follow-up duration (long-term, ≥ 1 year; short-term, ≤ 3 months; and immediate, ≤ 2 weeks). This study has been registered on PROSPERO (ID: CRD42018089605).

**Results:**

This meta-analysis included 13 studies (3 RCTs and 10 observational studies) and 8,351,815 subjects (1,124,672 cases and 7,227,143 controls). Overall, CLR use was not associated with increased long-term all-cause mortality (pooled rate ratio RR = 1.09, 95% CI = 0.91–1.32), either among patients with or without comorbidities of cardiovascular diseases. Comparing CLR users to placebo, there is no additional risks of cardiac mortality (pooled RR = 1.03, 95% CI = 0.53–2.01), acute myocardial infarction (pooled RR = 1.29, 95% CI = 0.98–1.68), and arrhythmia (pooled RR = 0.90, 95% CI = 0.62–1.32).

**Conclusions:**

Our findings suggested no significant association between CLR use and subsequent long-term all-cause mortality, regardless having comorbidity of cardiovascular diseases or not. Further RCTs investigating the short-term CV risks of CLR use compared to alternative antibiotics are warranted, particularly in high-risk populations.

## Introduction

According to the 2011 statistics of outpatient insurance database, there are more than 260 million antibiotic prescriptions per year in the United States. Among them, macrolides (e.g., clarithromycin (CLR), erythromycin, azithromycin, roxithromycin, etc.) are the most common antibiotics.[1] CLR has better bioavailability, gastrointestinal tolerance, and bactericidal activity than other macrolides,[2] supporting its broad use from respiratory to gastrointestinal tract infections, as well as *Helicobacter pylori* infection. Previous meta-analysis suggested the use of macrolides being associated with increased risks of arrhythmia or QT interval prolongation through multivariate models, possibly due to electrophysiological side effects.[3]

However, results from epidemiological studies using multivariate models regarding the cardiovascular (CV) risks associated with CLR use remained controversial because of different study designs and follow-up durations. One population-based study with a short-term follow-up period revealed a 2.2 times increased risk of arrhythmia among CLR users [4]; whereas the study published by Chou *et al*.[5] found no significant association between CLR use and the risk of arrhythmia within 30 days. Similarly, one large cohort study with a long-term follow-up period revealed an increased risk of acute myocardial infarction (MI) associated with CLR use [6], but the other studies didn’t.[4, 7]

In 2015, the CLARICOR trial showed an increased risk of all-cause mortality comparing CLR use versus placebo among patients with stable coronary artery disease (CAD).[8] According to this single trial, the US Food and Drug Administration issued a warning in February 2018 to remind cautious use of CLR in CAD patients.[9] At the same time, a meta-analysis of macrolides reported CLR users being associated with a 59% higher risk of acute MI compared to non-macrolide users.[3] However, that study did not consider the differential effects in patients with various underlying diseases and follow-up durations.

In physicians’ perspective, the risks of CLR use should be compared with alternative antibiotics use, but not placebo. Knowing the risks of CLR use among populations with different diseases or follow-up durations is crucial for clinical decisions and patient selection. Therefore, a meta-analysis addressing these issues would bring clinical values. The objective of this systematic review and meta-analysis is to summarize the association between CLR use and the short-term and long-term all-cause and cardiovascular mortality in different study populations.

## Materials and methods

### Protocol and registration

We conducted the protocol of this study according to the Preferred Reporting Items for Systemic review and Meta-Analysis Protocols (PRISMA-P) 2015 statement.[10] The present study has been registered with the International Prospective Registry of Systemic Reviews (PROSPERO CRD42018089605).

### Data sources, searches and selection criteria

We searched publications in humans from databases of PubMed, EMBASE, Web of Science, and the Cochrane Library until December 31^st^, 2018. A manual search of the reference lists was conducted to find additional studies. The Medical Subject Heading (MeSH) terms of “clarithromycin” and “cardiovascular disease” were used during the searching process, along with the entry terms and other keywords listed in the supporting information. Potential articles were screened manually by two independent reviewers (CHY, PHC) according to the titles and abstracts. Eligible study types included randomized controlled trials (RCTs) and observational studies (e.g., prospective or retrospective cohort studies, and case-control studies). Abstracts, letters to the editor, reviews, case reports, and self-controlled case series studies were excluded. Studies containing the following information were included: (1) exposure with CLR treatment versus either comparative antibiotics or placebo; (2) mortality or cardiovascular events as the outcome of interests; and (3) adult populations (≥ 18 years).

### Data extraction

Data from each enrolled study were extracted by two independent reviewers (CHY, PHC) to ensure quality and accuracy. Extracted variables included: author, journal, publication year, country, study design, sample size, underlying disease of subjects, exposure doses and durations of medication/placebo use, names of the comparator, follow-up durations, types of outcomes, and effect measures. Any disagreement in data extraction was discussed with a third reviewer until consensus was reached.

All-cause mortality was considered as primary outcome of interest because it is the most commonly reported endpoint. Acute myocardial infarction (AMI), cardiac mortality, and arrhythmia were considered as secondary outcomes. The definitions of arrhythmia in those enrolled studies consisted of a composite of atrioventricular conduction disorders, tachycardia and bradycardia accordingly. Follow-up duration was determined between the first date of antibiotic prescription and occurrence of outcome or censoring. One previous study used three months as the cut-off threshold for follow-up duration after CLR use.[11] Besides, the follow-up durations of the enrolled 13 study populations ranged from 5 days to 3 months, or 1 to 3 years. Thus, we divided the follow-up time into short-term (<3 months) and long-term (≥ 1 year). The immediate follow-up duration was defined as ≤ 2 weeks, since clarithromycin was rarely used for more than 2 weeks. The pooled effect estimates were calculated accordingly.

### Statistical analysis

Random effects models [12] were chosen *a priori* to calculate summary effect estimates, weighting for inverse variance separately for RCTs and observational studies. For studies reporting multiple types of prescriptions for CLR, we chose a one-time prescription to align with the study results. We used rate ratio (RR) as the main pooled effect estimates, considering the time-to-event effect; the alternative was risk ratio if rate ratio was not available. For the estimates by odds ratio or hazard ratio, we would reach out to the authors for raw data or convert them to rate ratio estimates using the following equations [13]:

$$\mathrm{RR}=\frac{\mathrm{OR}}{\left(1-r)+(r*\mathrm{OR}\right)};\;\mathrm{RR}=\frac{1-e^{\mathrm{HR}*\mathrm{LN}(1-r)}}{r}$$

where OR is odds ratio; LN is natural logarithm formula; RR is rate ratio; HR is hazard ratio; and r is the outcome event rate for the reference group.

We estimated the pooled risk of the primary outcome of interest (i.e., long-term all-cause mortality) based on the multivariate models in those studies, followed by the estimates in different subgroups (e.g., placebo or alternative antibiotics and with or without comorbidities) and follow-up durations. The pooled risks of secondary outcomes were further summarized by different follow-up durations and types of studies (i.e., RCT and observational studies) as well.

### Quality assessment

Quality assessment was performed by two reviewers independently. The Cochrane Collaboration’s tool [14] was used to assess the risk of bias in RCTs, including five metrics (six items): (1) adequacy of randomization, (2) allocation concealment, (3) blinding, (4) completeness of outcome data, and (5) selective reporting. Each item was graded as low, high, and unclear risk of bias. Studies without any item of high risk of bias were considered as high quality. For cohort and case-control studies, we used the Newcastle-Ottawa Quality Assessment Scale (NOS) [15], with a full score of 9, containing three subsections (9 items): (1) selection; (2) comparability; and (3) exposure (for case-control study) or outcome (for cohort studies). Studies with scores equal or greater than 6 points were considered as high quality.

Potential publication bias was evaluated by the Egger's regression test. The *I*^*2*^ test and Q statistics were also calculated to assess heterogeneity between studies. An *I*^*2*^ higher than 50% or *p*-value of the Q statistic less than 0.05 were considered as large heterogeneity.[16, 17] Leave-one-out sensitivity analyses were performed for effect estimates with large heterogeneity. The subgroup analyses were conducted by study types, quality of studies (NOS score), and effect measures (HR or not). All analyses were conducted using R version 3.6.1 and Stata version 15.0 (Stata Corporation, Texas, USA).

## Results

**Fig 1:**
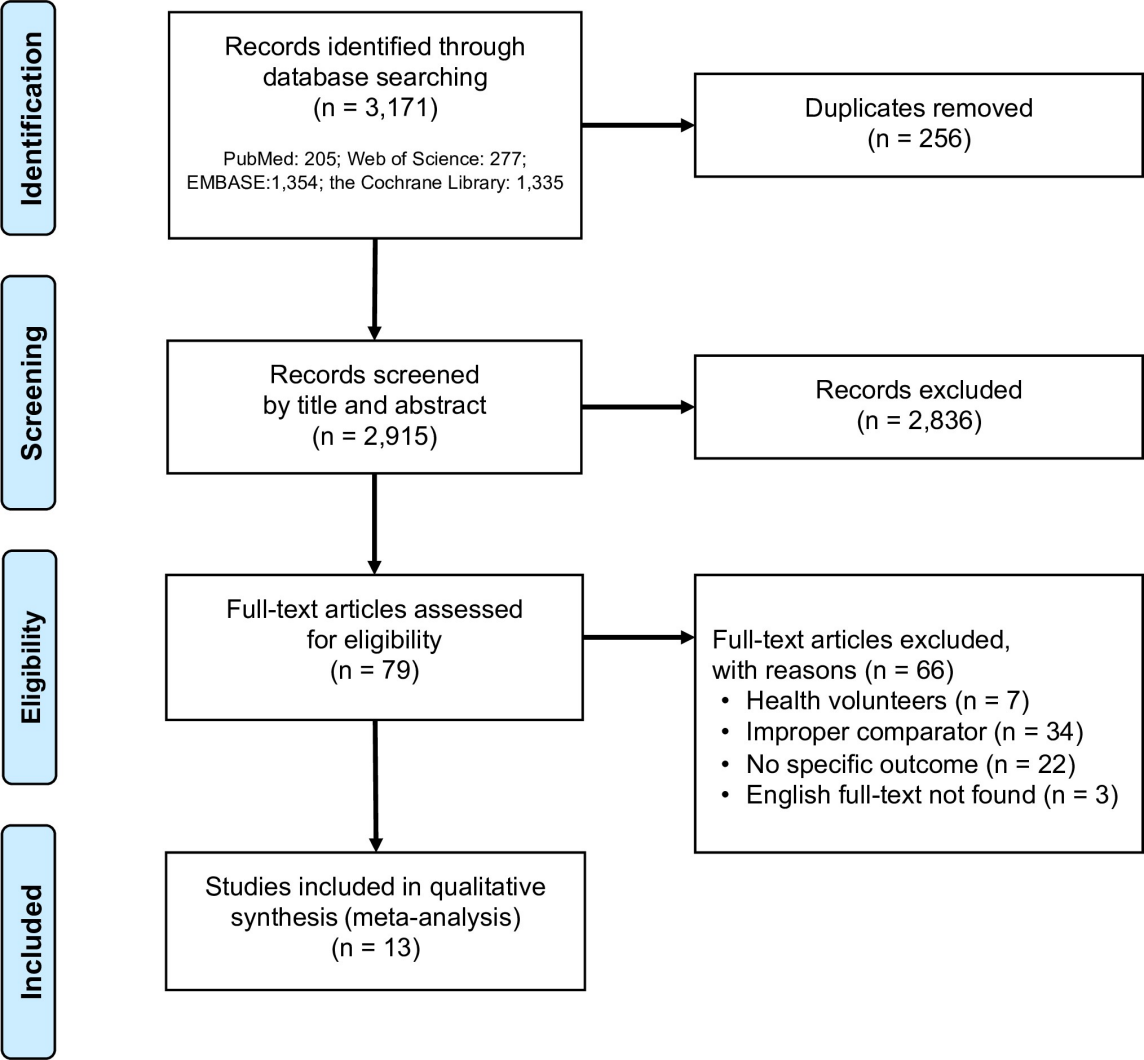
The flowchart of study enrollment.

Out of the 3,171 records from the initial screening, after excluding those not fit the inclusion criteria, 79 articles were reviewed for the full-text. The searching algorithm was presented in detail in the Fig 1.

This meta-analysis included three RCTs [8, 11, 18] and ten observational studies (nine cohorts [4–7, 19–23] and one nested case-control study [24]), with a total of 8,351,815 subjects (1,124,672 cases and 7,227,143 controls), as shown in Table 1. In RCTs, all participants had CAD before randomization, instead of acute bacterial infection, and these RCTs chose placebo as controls rather than alternative antibiotics. In ten observational studies, the study participants, either from the general population (8 studies) or having chronic comorbidities (2 studies, ischemic heart disease and chronic obstructive pulmonary disease), were indicated for antibiotic treatment for infectious diseases (e.g., pneumonia or *H*. *pylori* infection).[7, 22] Thus, they were compared to active comparator antibiotics, instead of placebo. Regarding to follow-up durations, short-term and long-term outcomes were reported in 7 and 5 observational studies, respectively. RCTs only reported long-term outcomes.

**Table 1:** Basic characteristics of 13 studies included in this meta-analysis.

Studies	Country	Sample size (Trt/ctrl)	Comparator antibiotic	Comorbidities of study population	Outcomes	Effect measures	Point estimates (95%CI)	Follow-up duration	
								Short-term (days)	Long-term (years)
**RCT**									
Sinisalo *et al*. (2002)	Finland	74/74	Placebo	Acute non–Q-wave infarction or unstable angina	AMI	Risk ratio	0.36 (0.14–0.94)		1.5
Berg *et al*. (2005)	Netherlands	238/235	Placebo	Before CABG surgery	ACM AMI	Rate ratio* Risk ratio	1.10 (0.45, 2.59) 0.33 (0.03, 3.14)		2 2
Winkel *et al*. (2015)	Denmark	2,172/2,200	Placebo	Stable coronary heart disease	ACM AMI	Rate ratio** Risk ratio	1.25 (1.04, 1.49) 0.97 (0.87, 1.09)		3 3
**Observational Study**^¶^									
Andersen *et al*. (2010)	Denmark	1,205/437	Non-CLR antibiotics	Ischemic heart disease	ACM	Rate ratio	1.07 (0.90, 1.26)		1
Hutson *et al*. (2012)^§^	Canada	59/295	AZM	General population (aged > 65 years)	AA	Risk ratio	0.65 (0.35, 1.23)	30	
Schembri *et al*. (2013)	UK	(COPD) 281/1,062 (CAP) 980/651	Non-CLR antibiotics	COPD and CAP	(COPD) ACM AMI	Rate ratio** Risk ratio	1.15 (0.90, 1.49) 1.56 (0.81, 3.03)		1 1
					(CAP) ACM AMI	Rate ratio** Risk ratio	1.12 (0.86, 1.48) 1.85 (0.87, 3.93)		1 1
Svanström *et al*. (2014)	Denmark	160,297/ 4,355,309	Penicillin V	General population	CD	Rate ratio	1.76 (1.08, 2.85) 1.06 (0.62, 1.82)	7 37	
Chou *et al*. (2015)	Taiwan	393,243/ 1,102,358	AMC	General population	CD CD AA	Rate ratio* Rate ratio* Risk ratio	0.49 (0.33, 0.71) 0.41 (0.29, 0.56) 0.72 (0.53, 0.96)	14 30 30	
Wong *et al*. (2016)	China	90,411/ 186,888	AMX	General population	ACM CD AA	Rate ratio Rate ratio Risk ratio	1.97 (1.83, 2.11) 1.67 (1.36, 2.06) 0.94 (0.59, 1.51)	14 14 30	
					ACM AMI	Rate ratio* Risk ratio	0.84 (0.80, 0.86) 0.99 (0.84, 1.18)		1 1
Mosholder *et al*. (2017)	UK	287,748/ 267,729	Doxycycline	General population	ACM AMI	Rate ratio** Risk ratio	1.25 (1.23, 1.28) 1.40 (1.33, 1.47)		3 3
Inghammer *et al*. (2017)	Denmark	187,887/ 751,543	Penicillin V	General population	CD ACM	Rate ratio Rate ratio	1.66 (0.98, 2.79) 1.06 (0.98, 1.16)	7	
									1
Sutton *et al*. (2017)	US	38,133/ 283,743	AZM	General population	CD	Rate ratio*	0.96 (0.38, 2.41)	5	
Berni *et al*. (2017)	UK	63,223/ 963,075	AMX	General population	ACM AA	Rate ratio* Risk ratio	1.35 (1.22, 1.49) 1.28 (1.12, 1.46)	37 37	

UK, United Kingdom; US, United States; ACM, all-cause mortality; CD, cardiac death; AMI, acute myocardial infarction; AA, arrhythmia alliance; Trt, treatment; Crtl, control; AMX, amoxicillin; CLR, clarithromycin; AMC, amoxicillin-Clavulanate; AZM, azithromycin; RCT, randomized controlled trial; AMI, acute myocardial infarction; COPD, chronic obstructive pulmonary disease; CAP, community acquired pneumonia

^**¶**^ Enrollees in all observational studies were indicated for either CLR or other antibiotics treatment due to infectious diseases (e.g. pneumonia and *Helicobacter pylori* infection).

§ Nested case-control study

* Conversion from odds ratio (OR) to rate ratio (RR) via the equation of RR = $\frac{OR}{\left(1-r)+(r*OR\right)}$, r is the outcome event rate for the comparator group^10^

** Conversion from hazard ratio (HR) to rate ratio (RR) via the equation of RR = $\frac{1-e^{HR*LN(1-r)}}{r}$, r is the outcome event rate for the comparator group

### Primary analysis

**Fig 2:**
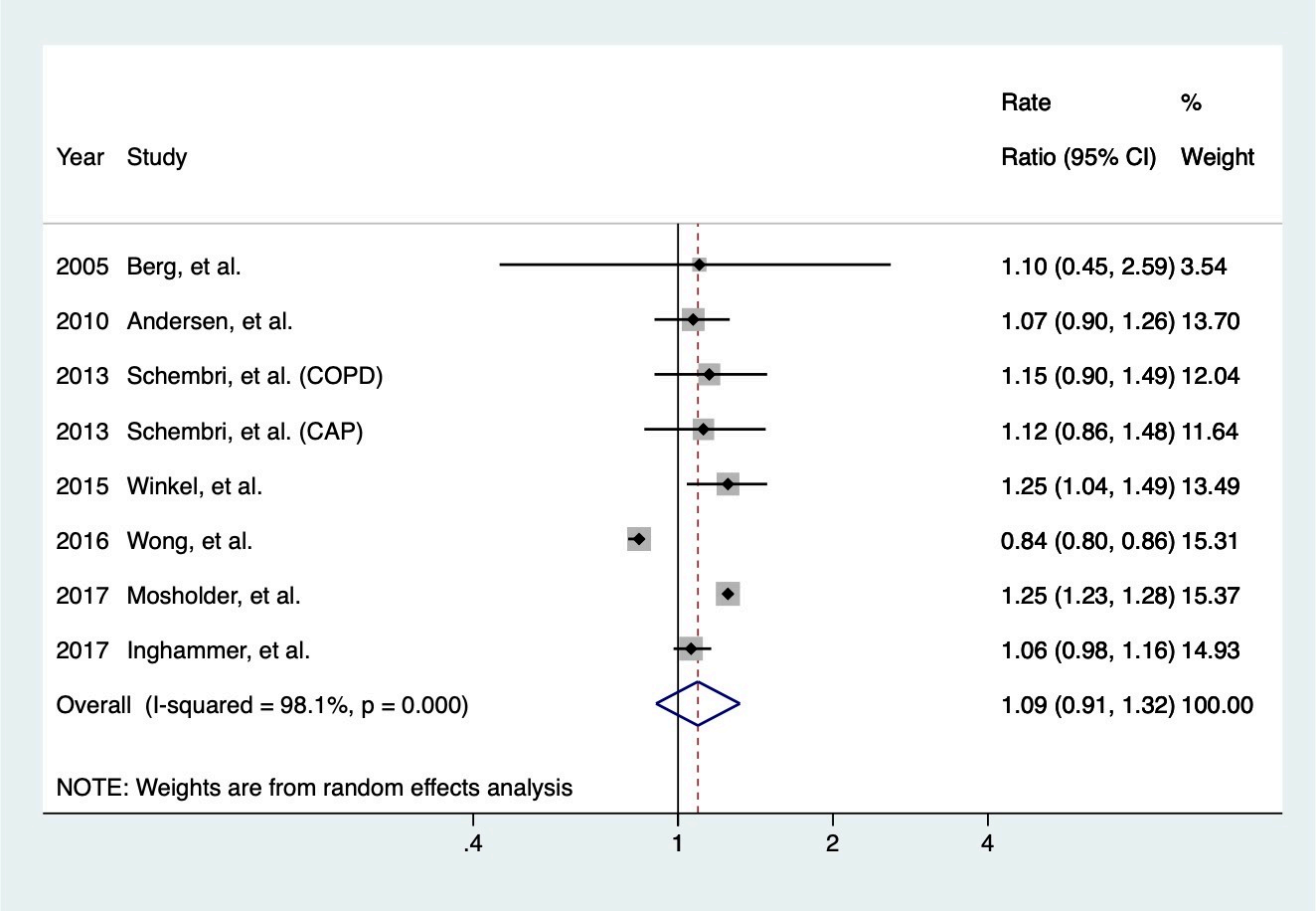
Primary analysis. The pooled rate ratios of all-cause mortality were summarized in studies with long-term (≥ 1 year) follow-up durations.

Meta-analysis combining RCTs and observational studies showed that CLR use was not associated with long-term all-cause mortality (pooled rate ratio RR = 1.09, 95% CI = 0.91–1.32) (Fig 2). Comparing CLR with other comparators, no higher risk of long-term all-cause mortality was found (pooled RR = 1.06, 95% CI = 0.94–1.20), regardless of patients with or without comorbidity of cardiovascular diseases (Table 2). However, with limited evidence (study number, N = 2), among patients with CAD, CLR use was associated with a 24% increased rate of all-cause mortality (pooled RR = 1.24, 95% CI = 1.04–1.48), compared to placebo. The short-term risk of all-cause mortality, yet with limited evidence (N = 2) as well, was associated with CLR use comparing to alternative antibiotics, among patients without comorbidity (pooled RR = 1.63, 95% CI = 1.13–2.37).

**Table 2:** Subgroup analysis. The pooled rate ratios of all-cause mortality were summarized by different subgroups.

Subgroups	N	Pooled rate ratios	*I*^*2*^	*p*-value
**Long-term effects**	8	1.09 (0.97–1.22)	93.72	0.154
CLR versus alternative antibiotics	6	1.06 (0.94–1.20)	95.34	0.330
- With comorbidities	3	1.10 (0.97–1.25)	0.00	0.135
- Without comorbidities	3	1.04 (0.82–1.30)	99.08	0.759
CLR versus placebo	2	1.24 (1.04–1.48)	0.00	0.015
**Short-term effects**				
CLR versus alternative antibiotics	2	1.63 (1.13–2.37)	97.26	0.090

CLR, clarithromycin.

### Secondary analysis

**Fig 3:**
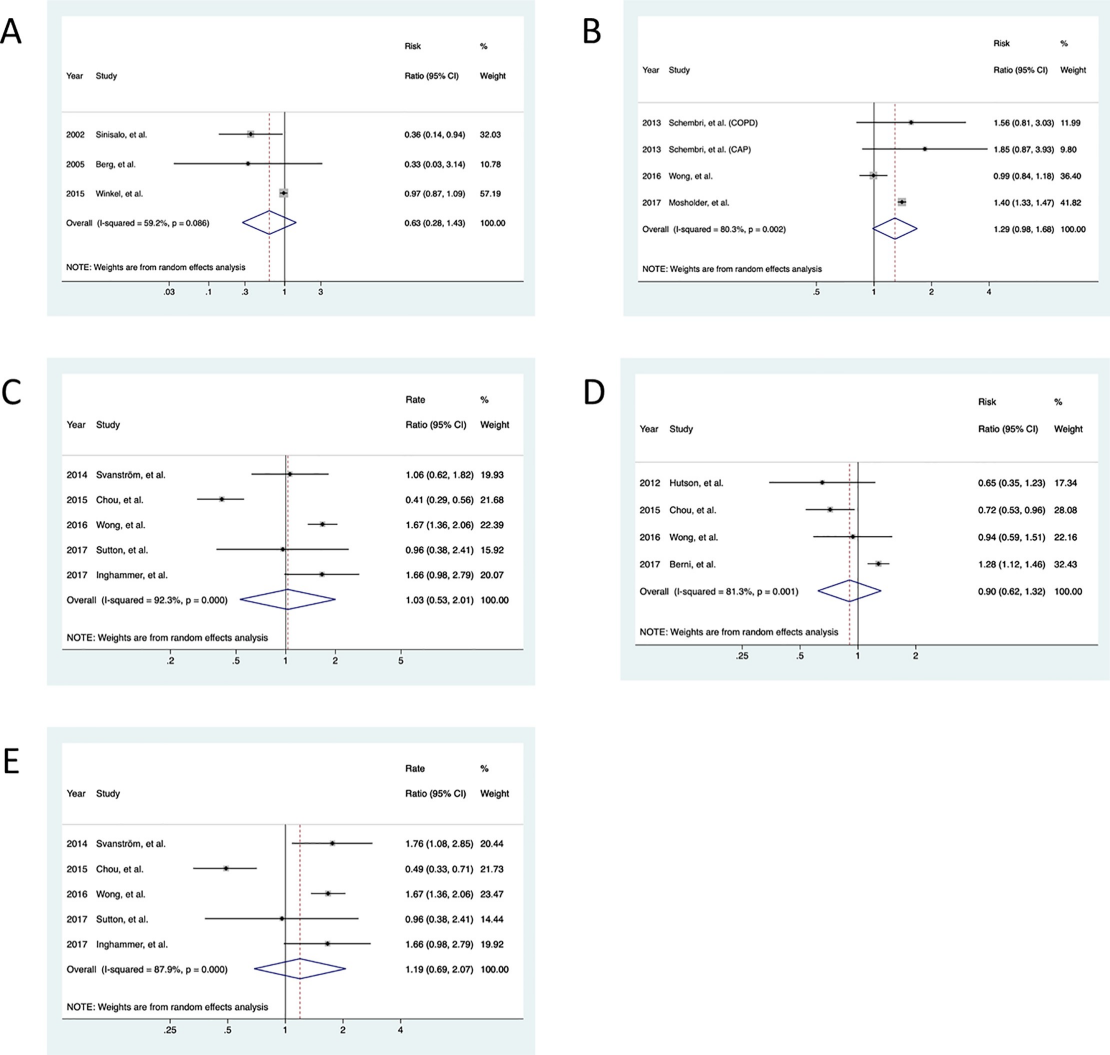
Secondary analysis. (A) The pooled risk ratios of acute myocardial infarction summarized in randomized controlled trials. (B) The pooled risk ratios of acute myocardial infarction summarized in observational studies with long-term (≥ 1 year) follow-up durations. (C) The pooled rate ratios of cardiac mortality in observational studies with short-term (≤ 3 months) follow-up durations. (D) The pooled risk ratios of arrhythmia in observational studies with short-term (≤ 3 months) follow-up durations. (E) The pooled rate ratios of cardiac mortality with immediate (≤ 2 weeks) follow-up durations.

There was no significant increased risk of any secondary outcomes for CLR use. Specifically, meta-analysis of three RCTs showed no increased risk of AMI comparing CLR versus placebo users (pooled RR = 0.63, 95% CI = 0.28–1.43) (Fig 3A). For observational studies, the summary estimate of five studies yielded no significant association between CLR use and increased risk of AMI after at least 1 year of follow-up (pooled RR = 1.29, 95% CI = 0.98–1.68) (Fig 3B). In terms of short-term cardiac mortality and arrhythmias, the pooled estimates of observational studies revealed a rate ratio of 1.03 (95% CI = 0.53–2.01) and a risk ratio of 0.90 (95% CI = 0.62–1.32), respectively, comparing CLR use versus alternative antibiotics use (Fig 3C and 3D). In subgroup analysis, CLR use was not associated with immediate risk of cardiac mortality (pooled RR = 1.19, 95% CI = 0.69–2.07) (Fig 3E), with a large heterogeneity (*I*^*2*^ = 87.9%, *P* < 0.001).

### Quality assessment and publication bias

The detailed quality assessments were summarized. By using the Cochrane Collaboration’s tool, three RCTs revealed low risks of bias, and ten observational studies had high qualities (S1 Fig). Egger’s regression test did not reach significance for publication bias in any of the analysis (all *P*-values > 0.05) (S1 Table and S2–S4 Figs). Leave-one-out sensitivity analyses also showed consistent and solid pooled estimates (S5 and S6 Figs). In subgroup analyses, the summary rate ratios of all-cause mortality were similar across different study types, study quality, and effect measures, although the heterogeneities were high in the subgroups of observational study (*I*^2^ = 98.4%) and non-HR group (*I*^2^ = 97.8%) (S2 Table).

## Discussion

In this meta-analysis, our results revealed no increased long-term all-cause mortality, comparing CLR and the other antibiotics, among patients with and without CAD. There was no increased risk of cardiac mortality, AMI, and arrhythmia, comparing CLR use versus alternative antibiotics use or placebo.

Our results suggested no significant increased long-term morality by CLR use in concordance with the lack of possible mechanistic explanation of long-term effects of CLR.[25] The potential explanation for macrolides related arrhythmia is by inhibiting potassium current in cardiac muscle cells,[26] which very unlikely persist for years. Most human studies were consistent with the observation that no delayed effects result in ischemic heart diseases or cardiovascular death one year after treatment starts.[4, 21, 23]

However, previous meta-analysis by Cheng *et al*.[27] in 2015 concluded elevated risks of CV events and all-cause mortality among CLR users compared with non-macrolide antibiotics users. Similarly, in 2018 another meta-analysis summarized an elevated risk of MI comparing CLR versus non-macrolide antibiotics use.[3] Both meta-analyses did not include updated researches and had some methodological problems. First, comparing to our study, eight and six relevant articles were not included for analyses in those meta-analyses by Cheng *et al*. and Gorelik *et al*., respectively. Second, without considering the differences of follow-up duration and study designs, previous analyses failed to appropriately address time window of the adverse effect. In fact, they improperly combined the results from short-term and long-term follow-up duration.[27] Third, the use of odds ratio in the meta-analysis did not account for the time-to-event effects, which may substantially affect the estimates according to our results.

In order to properly interpret the summary risk of CLR, the study design (placebo versus alternative antibiotics controls), study populations (with versus without cardiovascular diseases), and follow-up durations (long-term versus short-term), which may potentially bias the results, should be discussed. The rationales behind the two RCT studies comparing CLR and placebo were not reasonable at present.[8, 18] In fact, a fallacy in the early 1990s discussed that *Chlamydia pneumoniae* was the pathogen leading to atherosclerosis, and treating this pathogen by CLR may reduce risks of CAD.[28] Thus, the RCT performed at that time compared all-cause and CV mortality in CLR users to placebo, instead of alternative antibiotics users. Nowadays, CLR is mainly used to treat infectious diseases rather than CAD and thus investigating cardiovascular risks among patients of infectious diseases by comparing CLR with alternative antibiotic treatment is more reasonable.[29]

Interpretation of meta-analysis that included in the CLARICOR trial should be cautious for the following reasons.[8] First, the two-week duration of CLR exposure was much shorter than the follow-up duration of 10 years. There is no plausible biological explanation for the 10-year effects by a short-term use of rapid acting antibiotic, particularly when no increased risk of mortality and AMI was found within 3 years of follow-up.[30] This might suggest possible effects by other intermediates or factors after randomization. Second, only 32% of enrollees in the CLARICOR trial were randomized and there were imbalance factors (e.g., smoking) at baseline despite randomization, implying an invalid RCT design and potential biased results. Third, participants in the CLARICOR trial had no infectious disease indicated for CLR use, suggesting lack of generalizability to infection-diseased patients.

Our results from two cohort studies[4, 20] surprisingly showed that CLR users had a 63% increased rate ratio of all-cause mortality, but neither cardiac mortality nor arrhythmia, within 3 months of follow-up. The increased risk might result from non-cardiac mortality or potential bias by indication due to the cohort study design. By contrast, in 2017, one meta-analysis of cohort studies and RCTs revealed an increased risk of CV mortality, AMI, and arrhythmia among macrolides users after follow-up less than 30 days, but not in long-term follow-up.[25] In our results of observational studies, there was no increased risk of all-cause mortality and AMI among CLR users with follow-up greater or equal to 1 year, suggesting the biological effects of CLR may diminish as time went by. Similarly, in CLARICOR trial, the measures of risk by CLR use were increased during 0–3 years of follow-up and returned toward the null during 6–10 years of follow-up.[8] Given limited original studies investigating the short-term risks of CLR use so far, further clinical trials are warranted, particular in patients with cardiovascular diseases.

There are a few potential limitations in this study. First, the number of studies that met our inclusion criteria was small. The diverse study designs and study populations make it hard to simply summarize the effect measures. However, our methods are much comprehensive compared to previous meta-analyses. Second, there were large heterogeneities in this meta-analysis (*I*^*2*^ > 50%), possibly from various types of studies, selection of placebo or active comparators, and different baseline comorbidities. Thus, our subgroup findings provide more insights of risks of CLR users in different clinical settings.

## Conclusions

Comparing CLR and alternative antibiotics use, overall, our results showed no increased long-term all-cause mortality among patients with and without CAD. However, CLR may be associated with increased risks of all-cause mortality in specific populations or within a short-term of follow-up period. Further RCTs to compare CLR versus alternative antibiotics use in a short-term period or among patients with CV diseases were highly suggested.

## Supporting information

S1 File The Preferred Reporting Items for Systematic Reviews and Meta-Analyses (PRISMA) checklist. (DOC)

S2 File Supporting information of methods. Details in search keywords and quality assessment were provided.
(DOCX)

S1 Table Publication bias. The Egger’s tests showed no significant small study effects.
(DOCX)

S2 Table Subgroup analysis. The pooled rate ratios of all-cause mortality and heterogeneity were summarized by subgroups.
(DOCX)

S1 Fig Quality assessment. The risks of bias for randomized controlled trials and observational studies were assessed by using the Cochrane Collaboration’s tool and the Newcastle-Ottawa Quality Assessment Scale (NOS), respectively.
(TIF)

S2 Fig (A) Egger’s test and (B) Funnel plot of all studies on all-cause mortality with long-term follow-up. The results showed no obvious publication bias.
(TIF)

S3 Fig Egger’s tests of the observational studies. The results showed no obvious publication bias in studies with the outcomes of cardiac mortality after (A) short-term and (B) immediate follow-up durations and (C) studies with short-term outcome of arrhythmia.
(TIF)

S4 Fig Egger’s tests of those observational studies with long-term outcomes: (A) all-cause mortality and (B) acute myocardial infarction. The results showed no obvious publication bias.
(TIF)

S5 Fig Leave-one-out analysis of randomized controlled trials and observational studies with long-term outcomes of all-cause mortality revealed no strong effects by any single study. (TIF)

S6 Fig Leave-one-out analysis of observational studies with (A) short-term and (B) immediate outcomes of cardiac mortality showed no strong effects by any single study.
(TIF)
