# Supplementary material for: Clarithromycin use and the risk of mortality and cardiovascular events: A systematic review and meta-analysis
Source: PLoS One. 2019 Dec 27;14(12):e0226637. doi: 10.1371/journal.pone.0226637 (PMC6934307; doi:10.1371/journal.pone.0226637)

**Supporting information**

**SUPPORTING INFORMATION OF METHODS**

**Keywords used for literature search**

*MESH terms and keywords used for PubMed (equivalent for EMBASE, Web of Science, and the Cochrane Library)*

(Clarithromycin[mesh] OR clarithromycin[tiab]OR “6 o methylerythromycin”[tiab]OR “a 56268”[tiab]OR a56268[tiab]OR abbotic[tiab]OR “abbott 56268”[tiab]OR aeroxina[tiab]OR bactirel[tiab]OR biaxin[tiab]OR biclar[tiab]OR bicrolid[tiab]OR binoklar[tiab]OR bremon[tiab] OR “brevil od”[TIAB] OR c-clarin[tiab]OR carimycin[tiab]OR celex[tiab]OR clacin[tiab]OR clacine[tiab]OR clambiotic[tiab]OR clapharma[tiab]OR clari[tiab]OR claribid[tiab]OR claridar[tiab]OR clarikan[tiab]OR clarimac[tiab]OR claripen[tiab]OR clarith[tiab]OR claritrol[tiab]OR claroma[tiab]OR clormicin[tiab]OR crixan[tiab]OR cylind[tiab]OR cyllind[tiab]OR dicupal[tiab]OR “er 36469”[tiab]OR er36469[tiab] OR “erythromycin a, 6 o methyl”[tiab]OR gervaken[tiab]OR hecobac[tiab]OR heliclar[tiab]OR helitic[tiab]OR klacid[tiab]OR klacina[tiab]OR klaciped[tiab]OR klaribac[tiab]OR klaricid[tiab]OR klaridex[tiab]OR klaridia[tiab]OR klarin[tiab]OR klerimed[tiab]OR kofron[tiab]OR lagur[tiab] OR lekoklar[tiab] OR macladim[tiab]OR macladin[tiab]OR maclar[tiab]OR macrobiol[tiab]OR mavid[tiab]OR monozeclar[tiab]OR naxy[tiab] OR soriclar[tiab]OR “te 031”[tiab]OR te031[tiab]OR winclar[tiab] OR veclam[tiab]OR zeclar[tiab]) AND ("cardiovascular disease"[mesh] OR cardiovascular[tiab] OR angiocardiopathy[tiab] OR angiocardiovascular[tiab] OR heart[tiab] OR cardiac[tiab])

**Publication bias**

We used Egger’s test to examine publications bias among observational studies. The funnel plots for randomized control trials (RCTs) were thought to be non-informative due to limited study numbers. The funnel plots of the seven observational studies reporting short-term outcomes and of the five observational studies reporting long-term outcomes were observed symmetric (data not shown). The Egger’s tests also revealed no significant publication bias for short-term or long-term outcomes (*P* > 0.05). There was no sufficient evidence of small study effects among observational studies. (S1 Table)

**Quality assessment**

Quality assessment was done independently by two reviewers. The Cochrane Collaboration’s tool was used to assess the risk of bias in RCTs, including the following items as quality indicators: (1) adequacy of randomization, (2) allocation concealment, (3) blinding, (4) completeness of outcome data, and (5) selective reporting. Each item was graded as low, high, and unclear risk of bias. We used the Newcastle-Ottawa Quality Assessment Scale (NOS) for cohort and case-control studies. For cohort studies, eight quality indicators were used: (1) representativeness of the exposed cohort, (2) selection of the non-exposed cohort, (3) ascertainment of exposure, (4) incident disease, (5) comparability, (6) assessment of outcome, (7) length of follow-up, and (8) adequacy of follow-up. For case-control studies, eight quality indicators included (1) adequacy of the case definition, (2) representativeness of the cases, (3) selection of controls, (4) definition of controls, (5) comparability, (6) ascertainment of exposure, (7) ascertainment for cases and controls, and (8) non-response rate. Studies having scores equal to 7 points or more were considered as representing at low risk of bias. Disagreements were resolved by discussion with a third reviewer to reach consensus.

**Quality assessment of the enrolled studies**

Among the three RCTs, the authors properly avoided potential selection bias, performance bias, attrition bias, and reporting bias. The quality of these RCTs was overall good by using the Cochrane Collaboration’s tool assessment. (S1 Fig) After NOS evaluation, the ten observational studies were of good quality with a score of 6 or more. A common limitation was that they did not have enough (≥ 80%) of participants to follow up, so we chose the shortest follow-up time of longer than 1 year as the definition for long-term follow-up duration.

**S1 Table.** **Publication bias.** The Egger’s tests showed no significant small study effects.

| **Outcome** | **Study** | **Study**  **number** | **Bias term coefficient** | ***P*-value** |
| --- | --- | --- | --- | --- |
| **Primary Analysis** |  |  |  |  |
| **All-cause mortality** | RCT and long-term observational | 8 | 0.28 | 0.94 |
|  | RCT | 2 | -0.37 | NA |
|  | Short-term observational | 2 | -25.74 | NA |
|  | Long-term observational | 6 | -3.12 | 0.59 |
| **Secondary Analysis** |  |  |  |  |
| **Acute myocardial infarction** | RCT | 3 | -1.46 | 0.31 |
|  | Long-term observational | 4 | -0.71 | 0.74 |
| **Cardiac mortality** | Short-term observational | 5 | -1.85 | 0.67 |
| **Cardiac mortality (**$\boldsymbol{\leq}$**2 weeks)** | Short-term observational | 5 | -1.72 | 0.62 |
| **Arrhythmia** | Short-term observational | 4 | -3.05 | 0.16 |

RCT, randomized controlled trials; NA, non-applicable due to study numbers

**S2 Table.** **Subgroup analysis.** The pooled rate ratios of all-cause mortality and heterogeneity were summarized by subgroups.

| **Subgroup** | **Study**  **number** | **RR (95% CI)** | ***I*^2^ (%)** |
| --- | --- | --- | --- |
| **Study type** |  |  |  |
| **RCT** | 2 | 1.24 (1.04 – 1.48) | 0 |
| **Observational study** | 8 | 1.20 (1.01 – 1.42) | 98.4 |
| **NOS score** |  |  |  |
| $\boldsymbol{\geq}$ **7** | 8 | 1.21 (1.01 – 1.45) | 98.4 |
| $\boldsymbol{\geq}$ **8** | 4 | 1.25 (1.23 – 1.28) | 0 |
| $\boldsymbol{\geq}$ **9** | 3 | 1.32 (1.21 – 1.44) | 0 |
| **Effect measurement** |  |  |  |
| **Hazard ratio (HR)** | 4 | 1.26 (1.23 – 1.28) | 0 |
| **Non-HR** | 6 | 1.20 (0.94 – 1.53) | 97.8 |
| RCT, randomized controlled trials; RR, rate ratio; NOS, Newcastle-Ottawa quality assessment scale | | | |

**S1 Fig.** **Quality assessment.** The risks of bias for randomized controlled trials and observational studies were assessed by using the Cochrane Collaboration’s tool and the Newcastle-Ottawa Quality Assessment Scale (NOS), respectively.
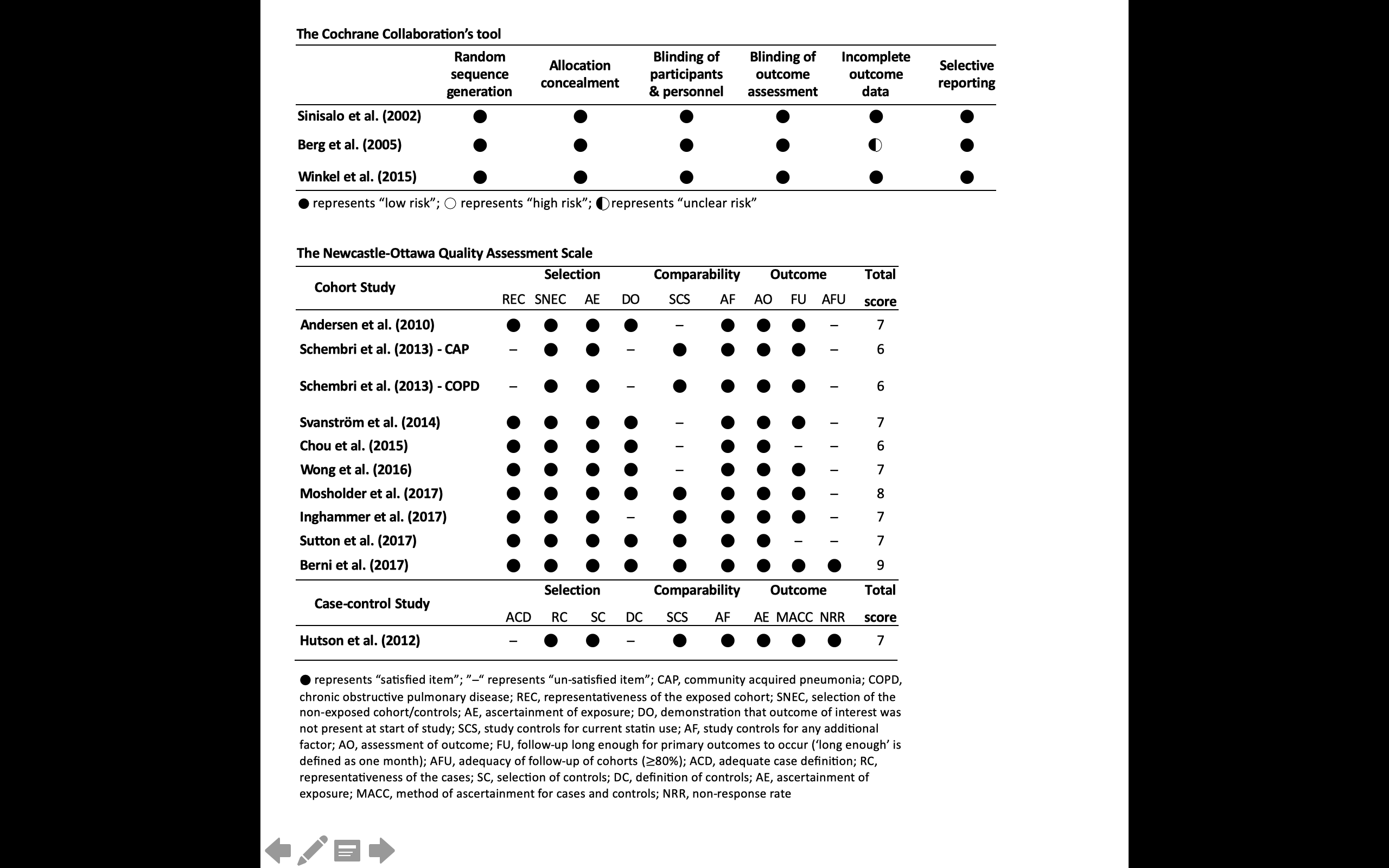


**S2 Fig.** (A) Egger’s test and (B) Funnel plot of all studies on all-cause mortality with long-term follow-up. The results showed no obvious publication bias.

**S2A Fig.**


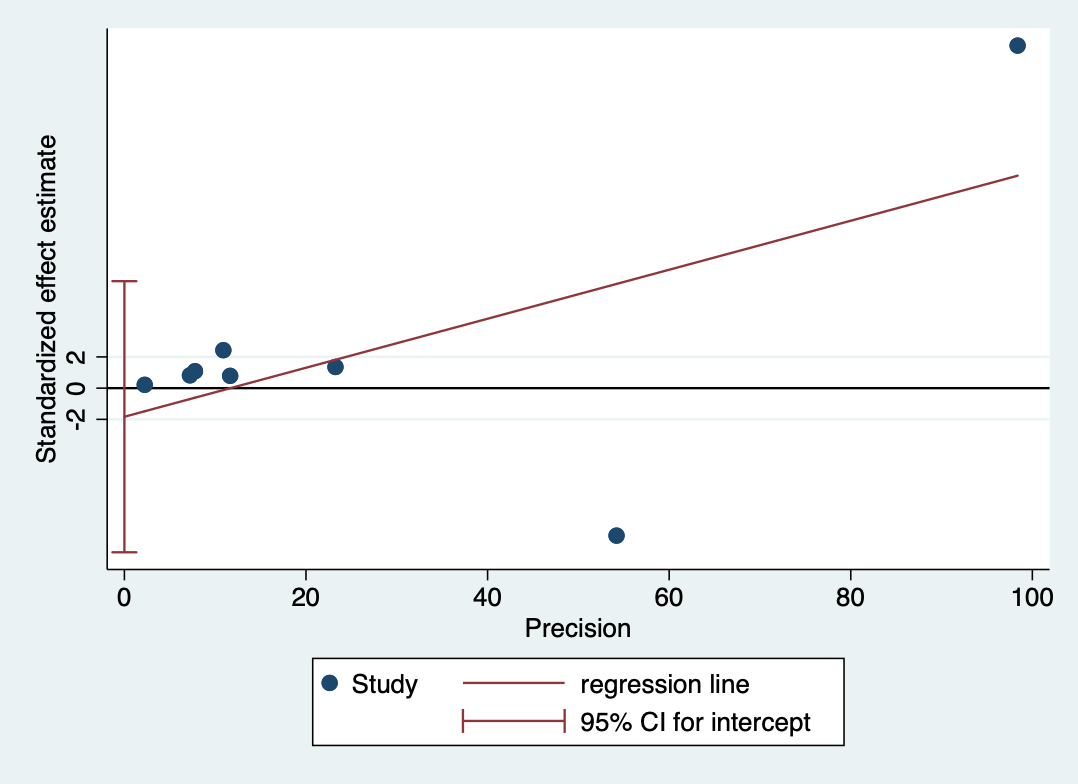


**S2B Fig.**

**
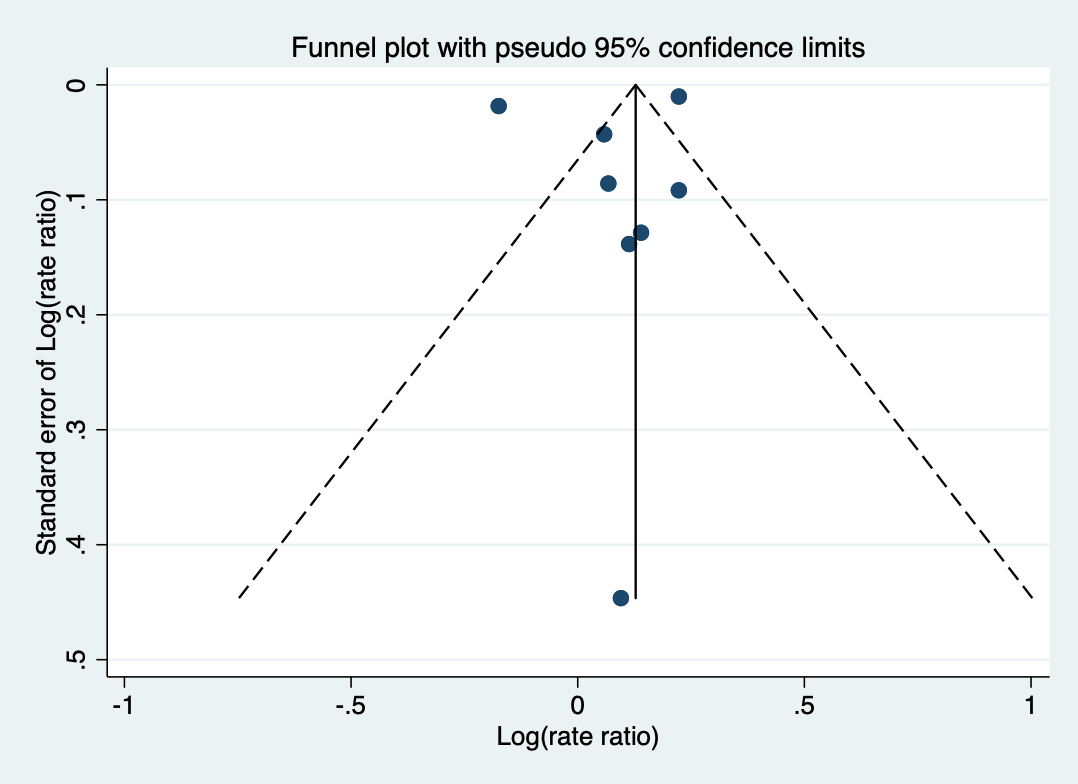
**

**S3 Fig.** **Egger’s tests of the observational studies.** The results showed no obvious publication bias in studies with the outcomes of cardiac mortality after (A) short-term and (B) immediate follow-up durations and (C) studies with short-term outcome of arrhythmia.

**S3A Fig.**


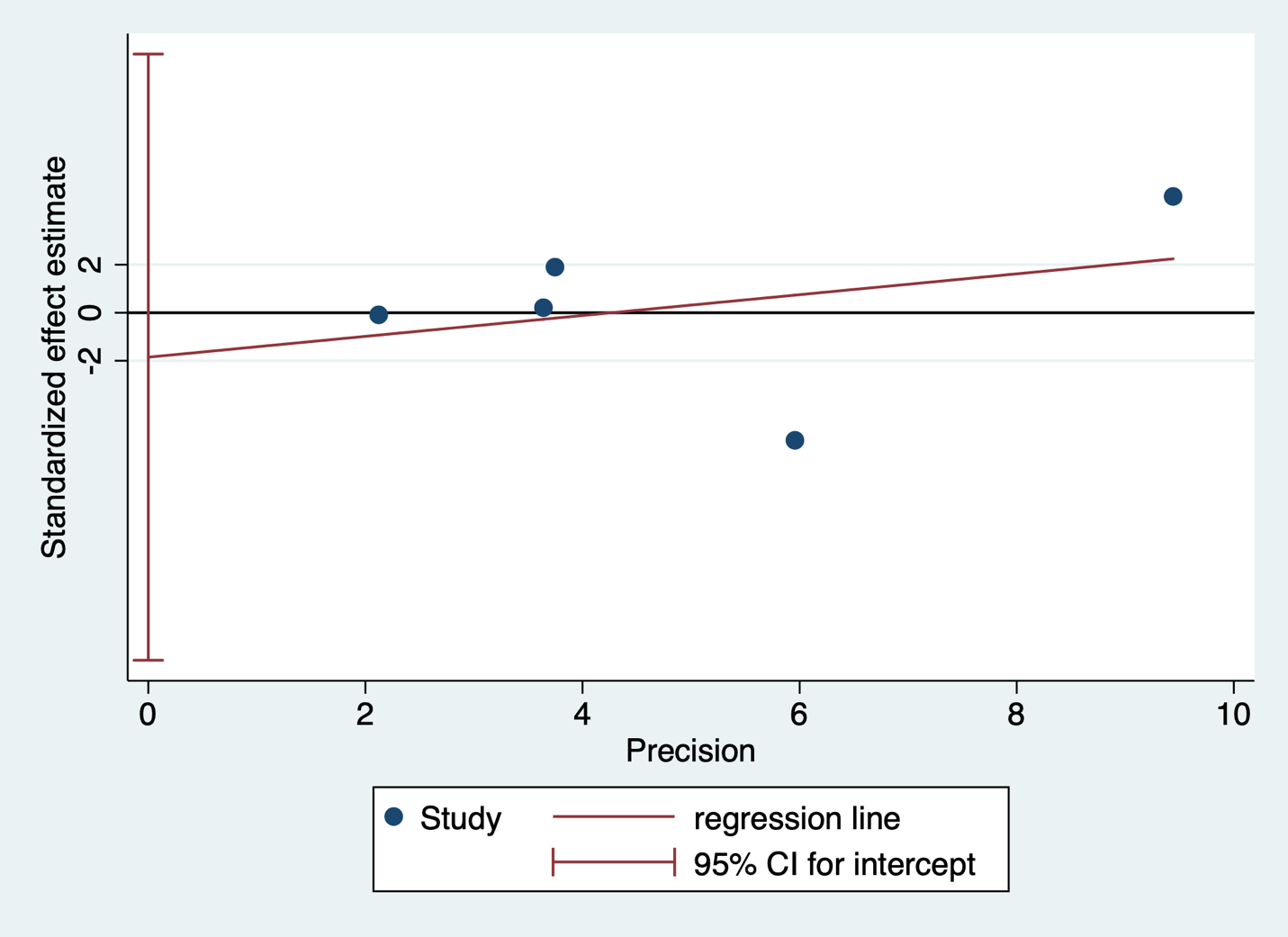


**S3B Fig.**


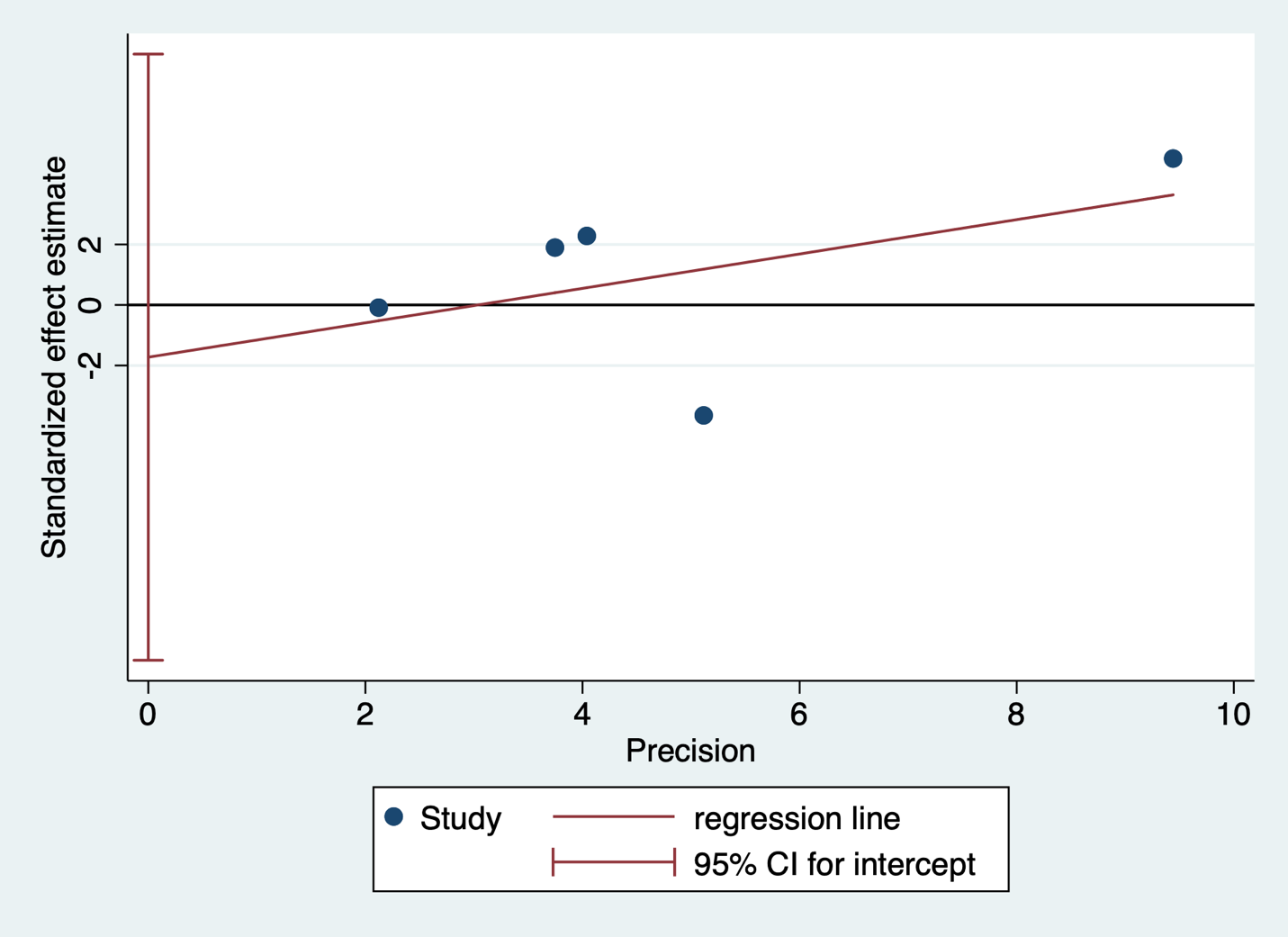


**S3C Fig.**


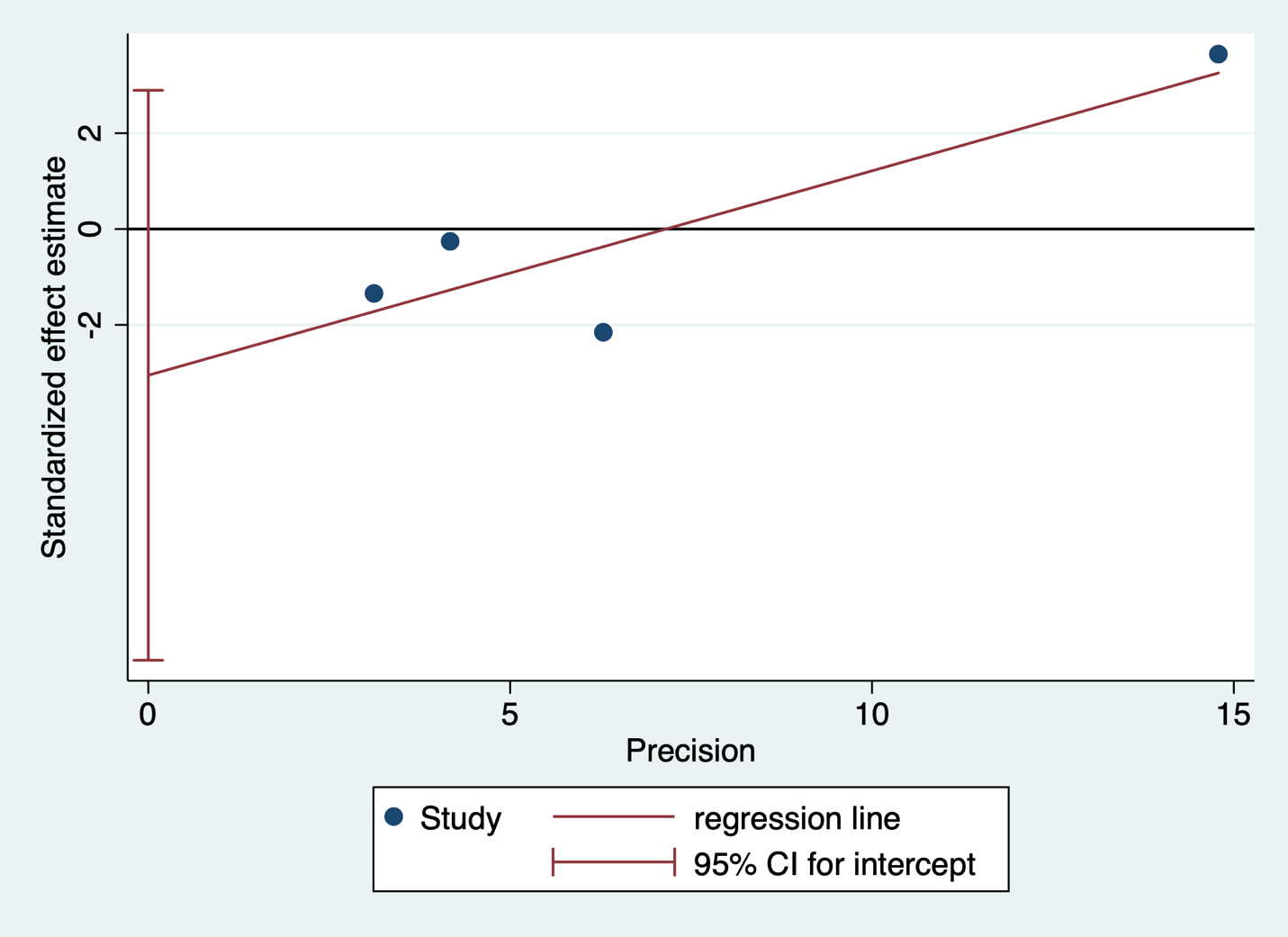


**S4 Fig.** Egger’s tests of those observational studies with long-term outcomes: (A) all-cause mortality and (B) acute myocardial infarction. The results showed no obvious publication bias.

**S4A Fig.**


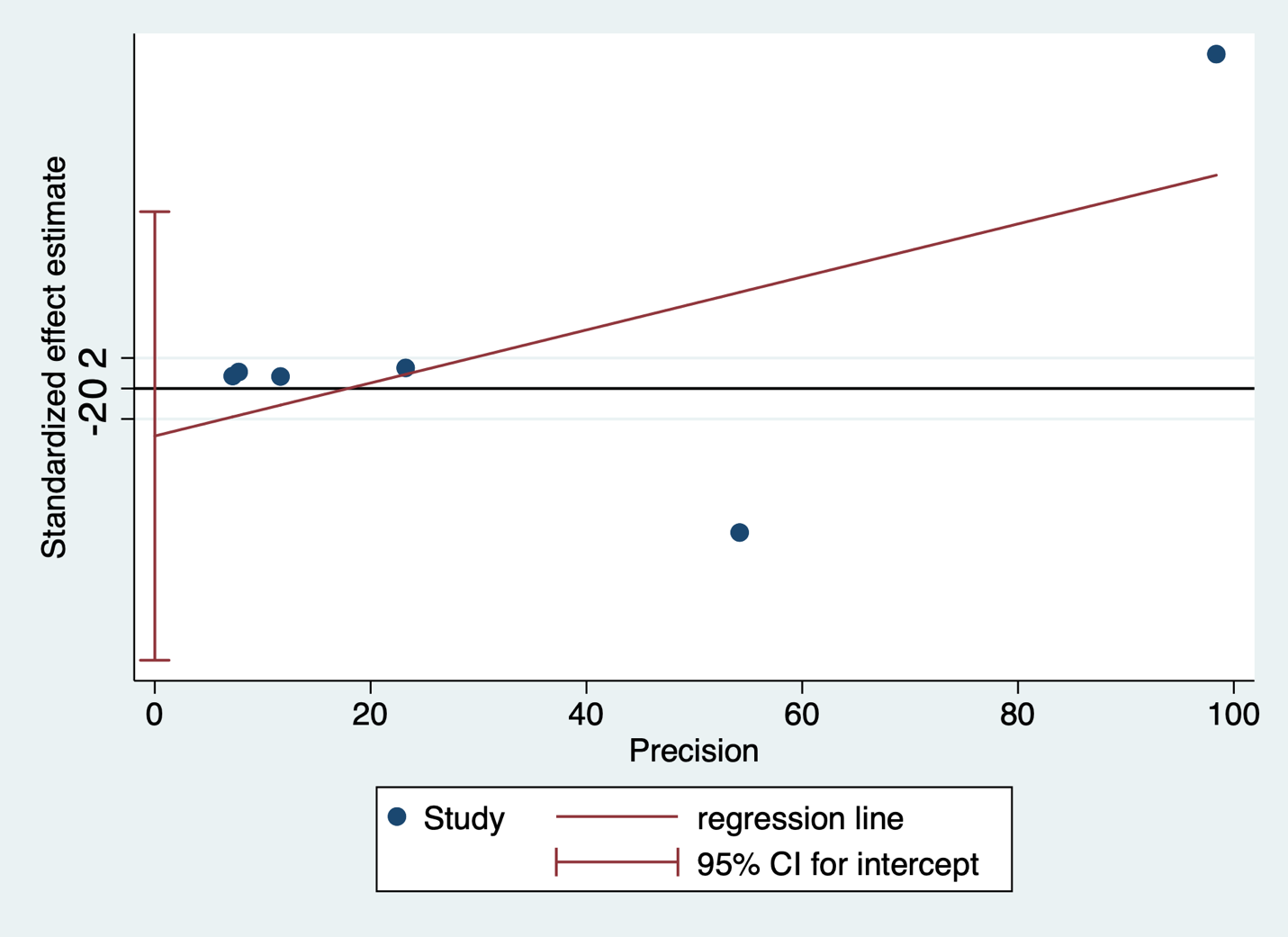


**S4B Fig.**


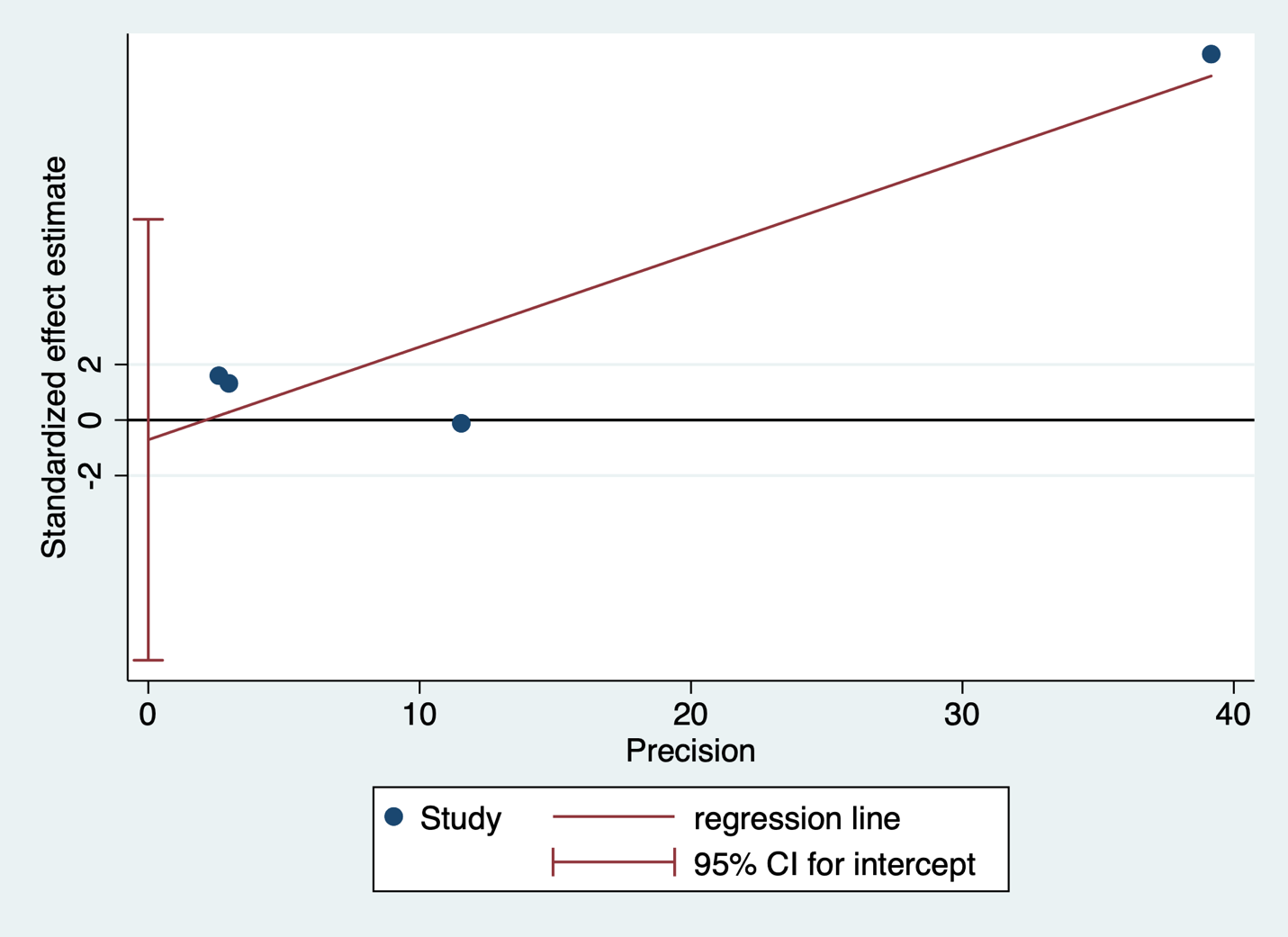


**S5 Fig.** Leave-one-out analysis of randomized controlled trials and observational studies with long-term outcomes of all-cause mortality revealed no strong effects by any single study.

**
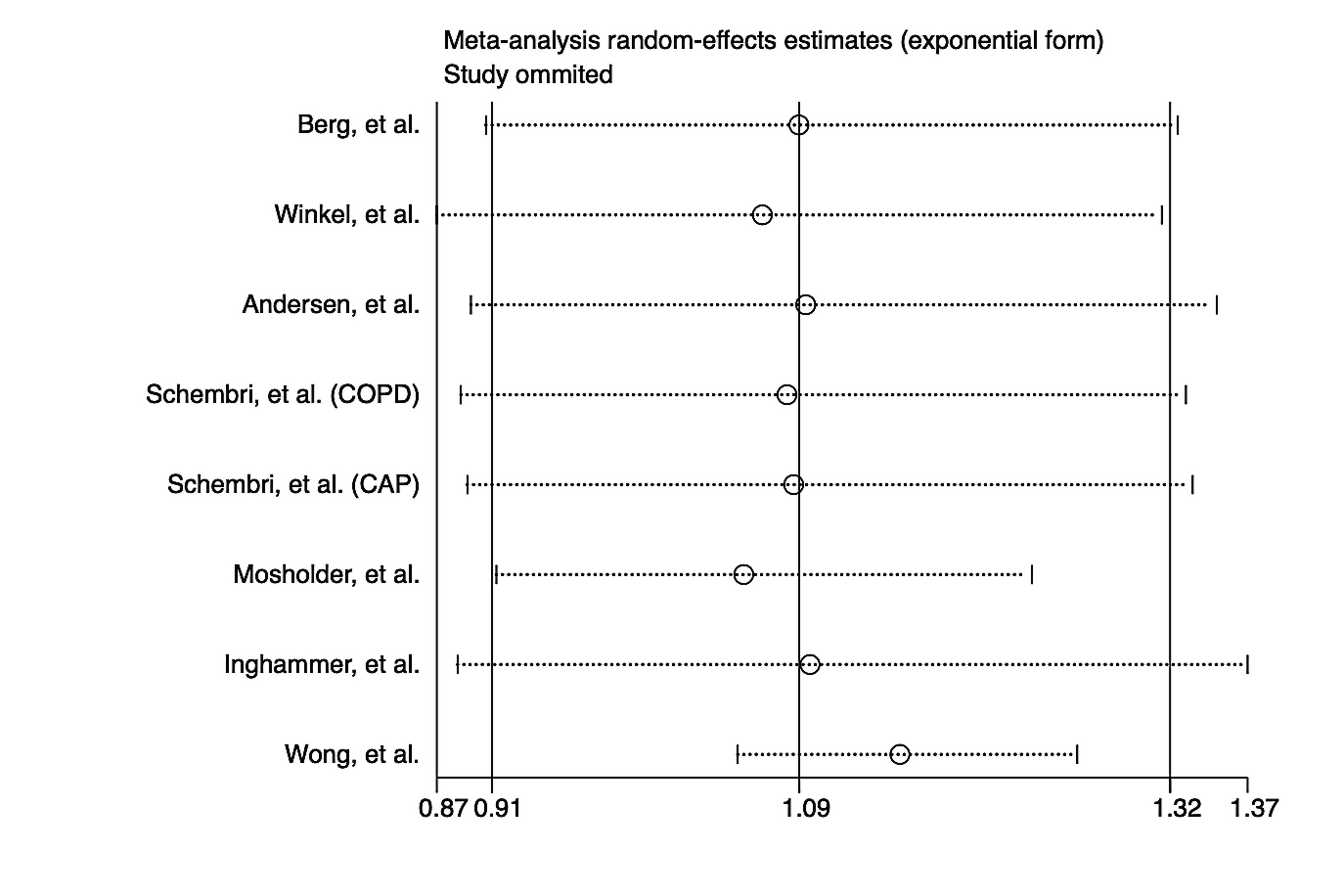
**

**S6 Fig.** Leave-one-out analysis of observational studies with (A) short-term and (B) immediate outcomes of cardiac mortality showed no strong effects by any single study.

**S6A Fig.**

**
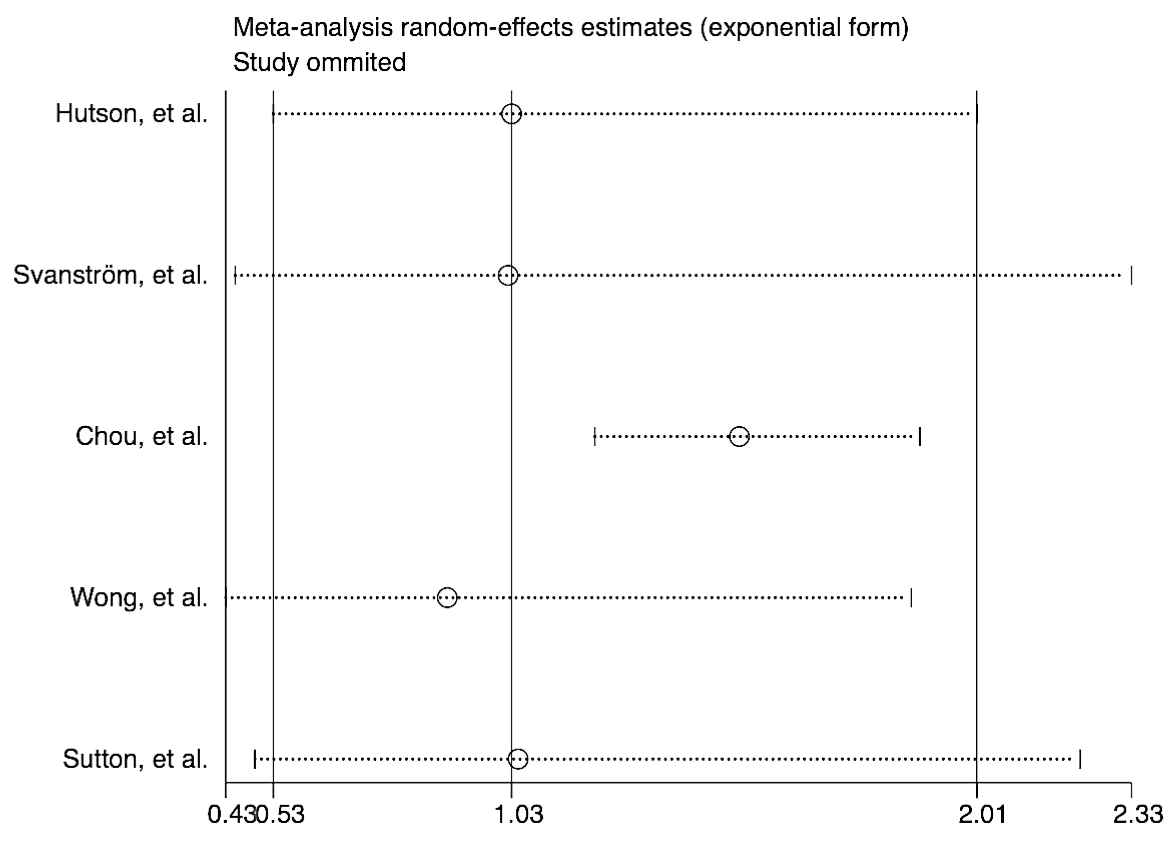
**

**S6B Fig.**


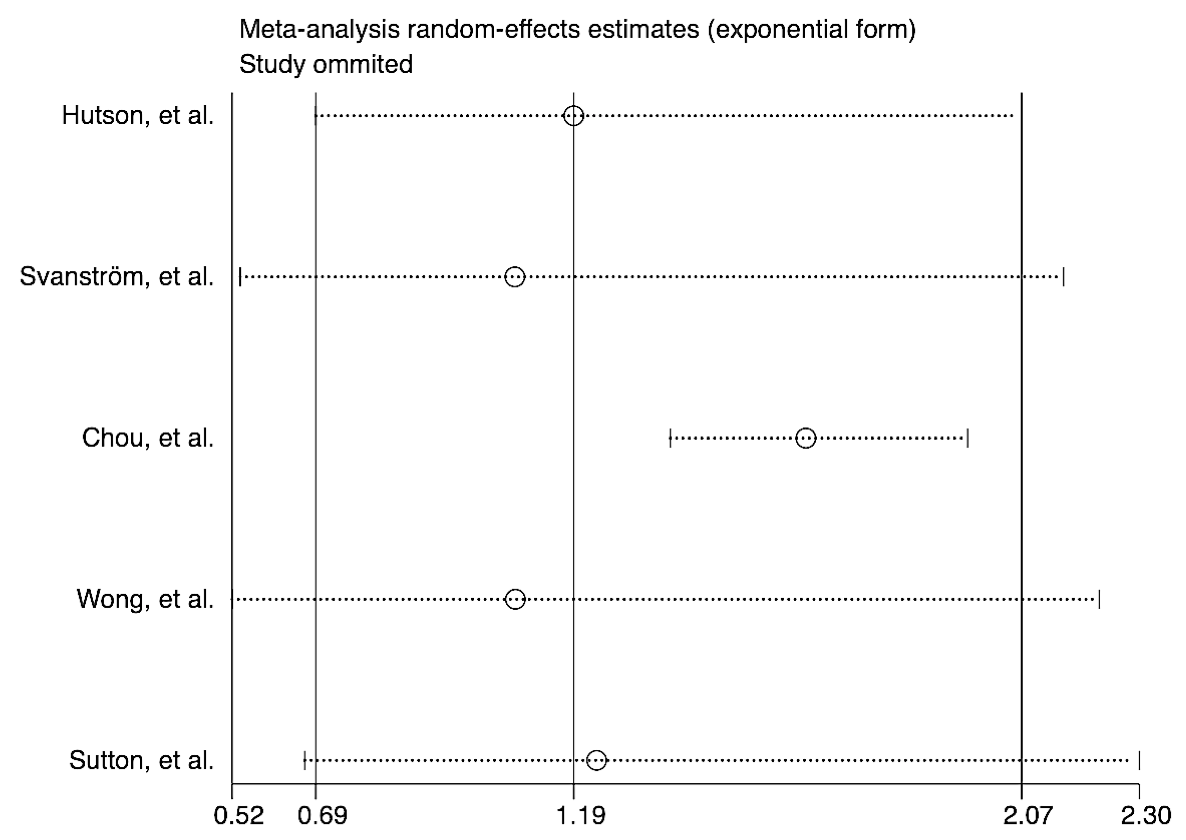

Supplement: S2 File — Details in search keywords and quality assessment were provided. (DOCX) [file pone.0226637.s002.docx]
