# Supplementary material for: Clarithromycin use and the risk of mortality and cardiovascular events: A systematic review and meta-analysis
Source: PLoS One. 2019 Dec 27;14(12):e0226637. doi: 10.1371/journal.pone.0226637 (PMC6934307; doi:10.1371/journal.pone.0226637)
Supplement: S1 Table — The Egger’s tests showed no significant small study effects. (DOCX) [file pone.0226637.s003.docx]

**S1 Table.** **Publication bias.** The Egger’s tests showed no significant small study effects.

| **Outcome** | **Study** | **Study**  **number** | **Bias term coefficient** | ***P*-value** |
| --- | --- | --- | --- | --- |
| **Primary Analysis** |  |  |  |  |
| **All-cause mortality** | RCT and long-term observational | 8 | 0.28 | 0.94 |
|  | RCT | 2 | -0.37 | NA |
|  | Short-term observational | 2 | -25.74 | NA |
|  | Long-term observational | 6 | -3.12 | 0.59 |
| **Secondary Analysis** |  |  |  |  |
| **Acute myocardial infarction** | RCT | 3 | -1.46 | 0.31 |
|  | Long-term observational | 4 | -0.71 | 0.74 |
| **Cardiac mortality** | Short-term observational | 5 | -1.85 | 0.67 |
| **Cardiac mortality (**$\boldsymbol{\leq}$**2 weeks)** | Short-term observational | 5 | -1.72 | 0.62 |
| **Arrhythmia** | Short-term observational | 4 | -3.05 | 0.16 |

RCT, randomized controlled trials; NA, non-applicable due to study numbers
