# Supplementary material for: Clarithromycin use and the risk of mortality and cardiovascular events: A systematic review and meta-analysis
Source: PLoS One. 2019 Dec 27;14(12):e0226637. doi: 10.1371/journal.pone.0226637 (PMC6934307; doi:10.1371/journal.pone.0226637)
Supplement: S2 Table — The pooled rate ratios of all-cause mortality and heterogeneity were summarized by subgroups. (DOCX) [file pone.0226637.s004.docx]

**S2 Table.** **Subgroup analysis.** The pooled rate ratios of all-cause mortality and heterogeneity were summarized by subgroups.

| **Subgroup** | **Study**  **number** | **RR (95% CI)** | ***I*^2^ (%)** |
| --- | --- | --- | --- |
| **Study type** |  |  |  |
| **RCT** | 2 | 1.24 (1.04 – 1.48) | 0 |
| **Observational study** | 8 | 1.20 (1.01 – 1.42) | 98.4 |
| **NOS score** |  |  |  |
| $\boldsymbol{\geq}$ **7** | 8 | 1.21 (1.01 – 1.45) | 98.4 |
| $\boldsymbol{\geq}$ **8** | 4 | 1.25 (1.23 – 1.28) | 0 |
| $\boldsymbol{\geq}$ **9** | 3 | 1.32 (1.21 – 1.44) | 0 |
| **Effect measurement** |  |  |  |
| **Hazard ratio (HR)** | 4 | 1.26 (1.23 – 1.28) | 0 |
| **Non-HR** | 6 | 1.20 (0.94 – 1.53) | 97.8 |
| RCT, randomized controlled trials; RR, rate ratio; NOS, Newcastle-Ottawa quality assessment scale | | | |
