# Supplementary figures and images for: Clarithromycin use and the risk of mortality and cardiovascular events: A systematic review and meta-analysis
Source: PLoS One. 2019 Dec 27;14(12):e0226637. doi: 10.1371/journal.pone.0226637 (PMC6934307; doi:10.1371/journal.pone.0226637)

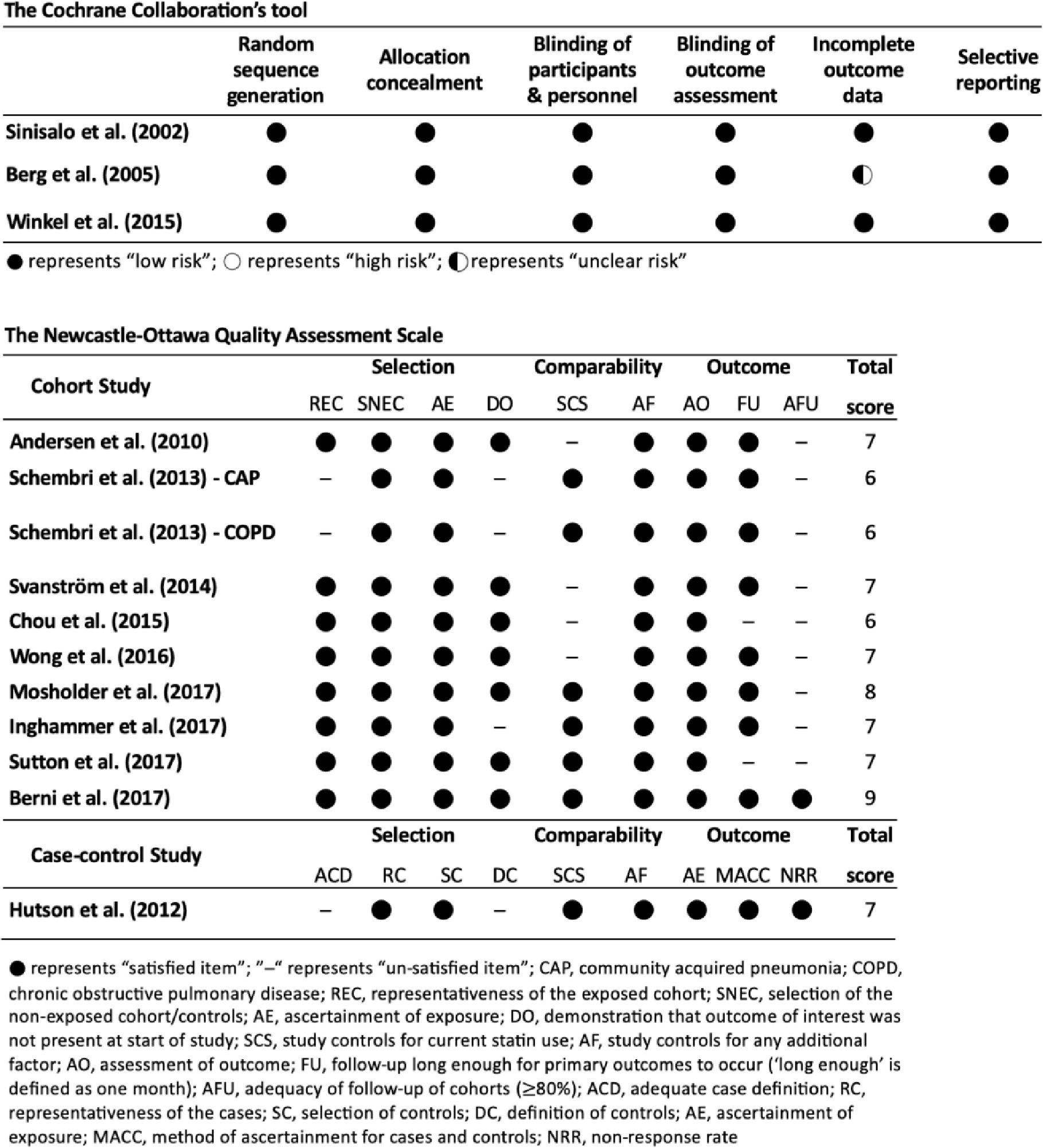

Supplement: S1 Fig — The risks of bias for randomized controlled trials and observational studies were assessed by using the Cochrane Collaboration’s tool and the Newcastle-Ottawa Quality Assessment Scale (NOS), respectively. (TIF) [file pone.0226637.s005.tif]

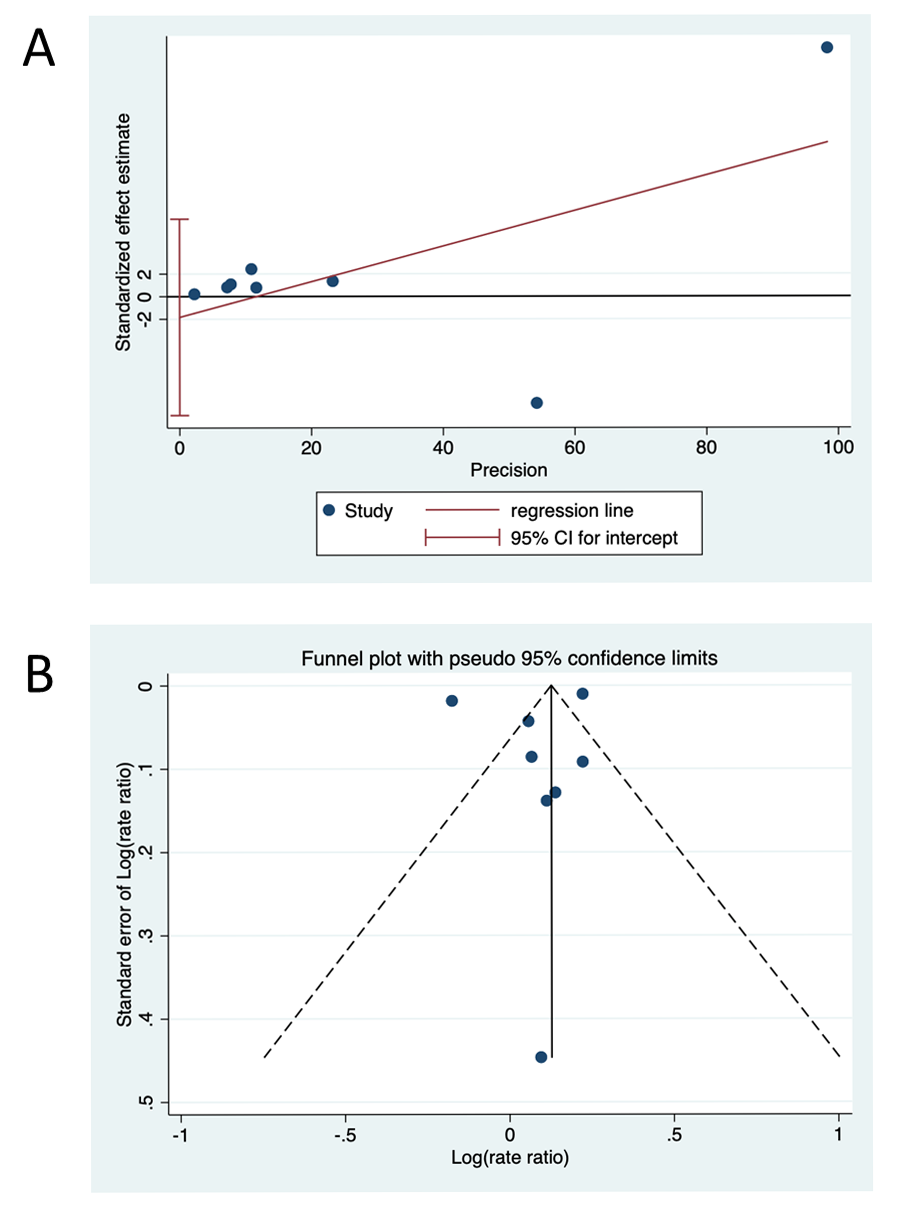

Supplement: S2 Fig — (A) Egger’s test and (B) Funnel plot of all studies on all-cause mortality with long-term follow-up. The results showed no obvious publication bias. (TIF) [file pone.0226637.s006.TIF]

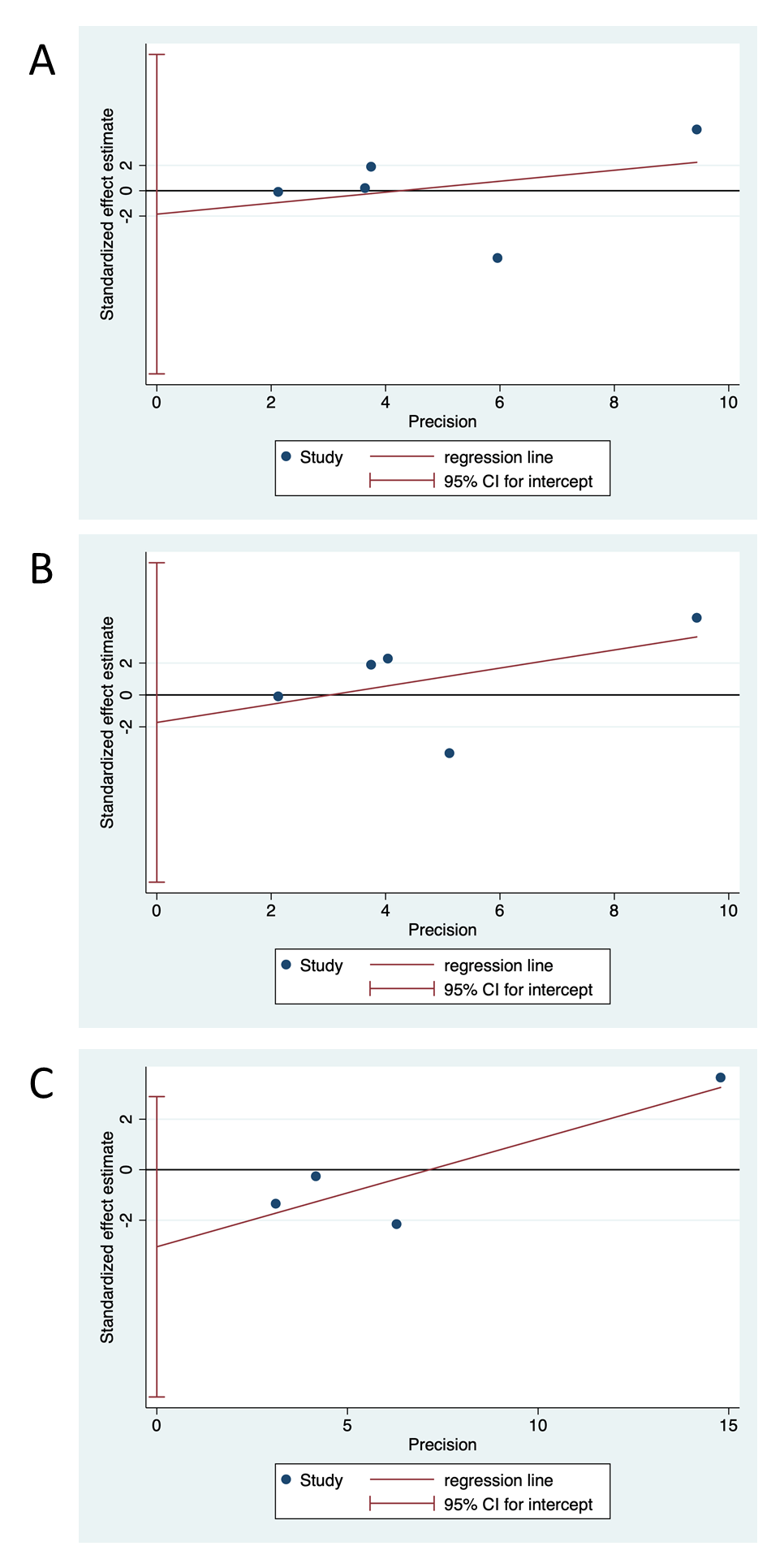

Supplement: S3 Fig — The results showed no obvious publication bias in studies with the outcomes of cardiac mortality after (A) short-term and (B) immediate follow-up durations and (C) studies with short-term outcome of arrhythmia. (TIF) [file pone.0226637.s007.TIF]

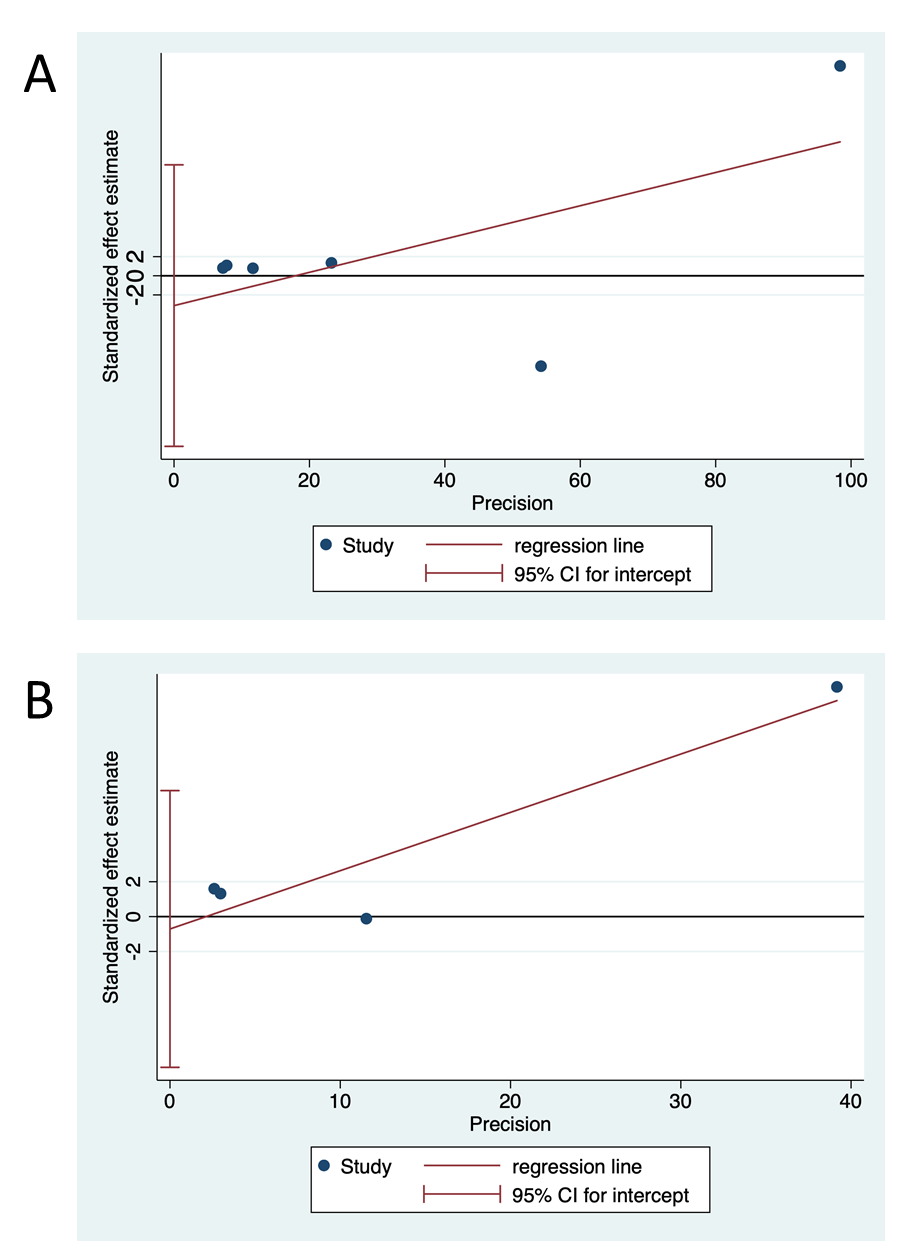

Supplement: S4 Fig — Egger’s tests of those observational studies with long-term outcomes: (A) all-cause mortality and (B) acute myocardial infarction. The results showed no obvious publication bias. (TIF) [file pone.0226637.s008.TIF]

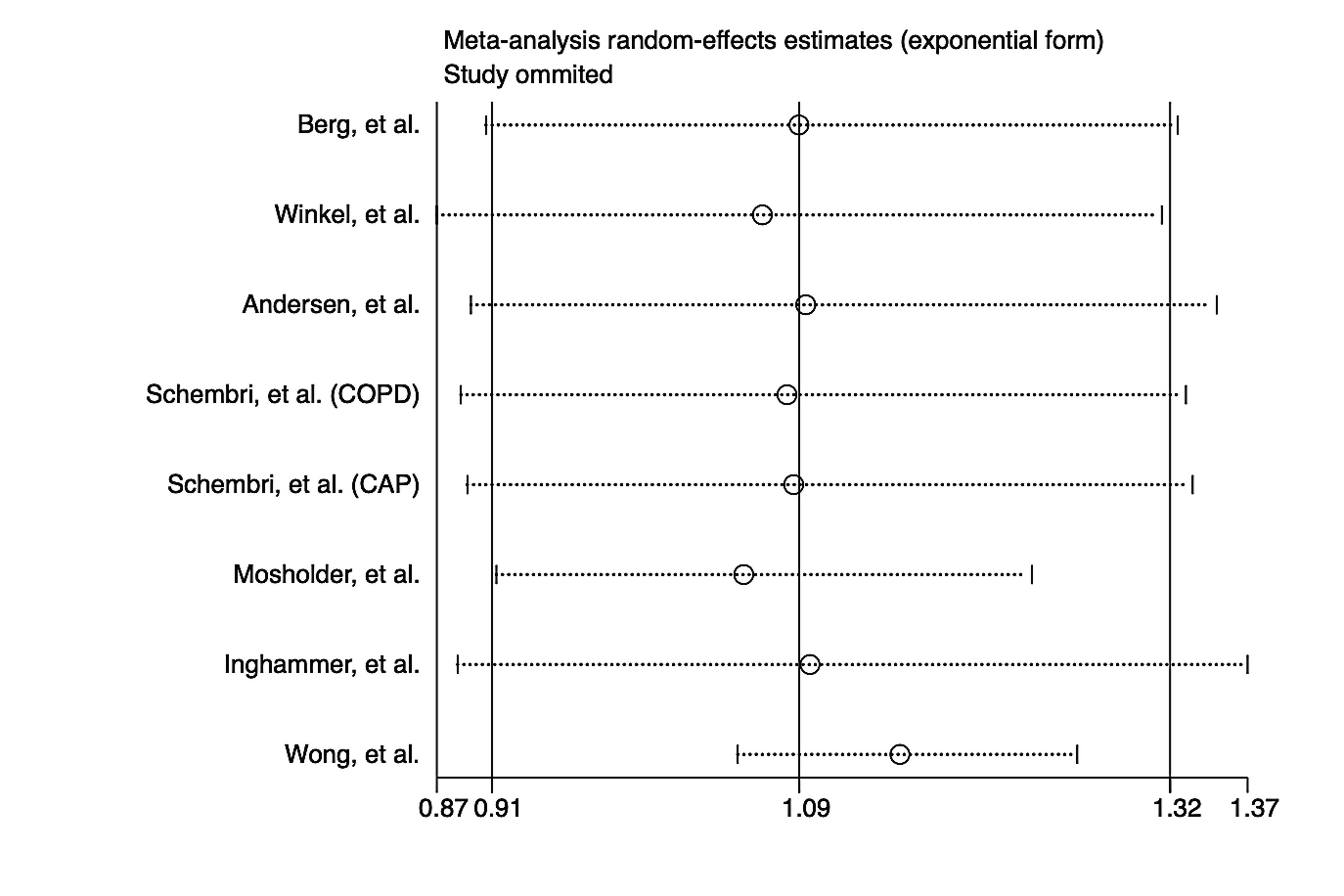

Supplement: S5 Fig — (TIF) [file pone.0226637.s009.tif]

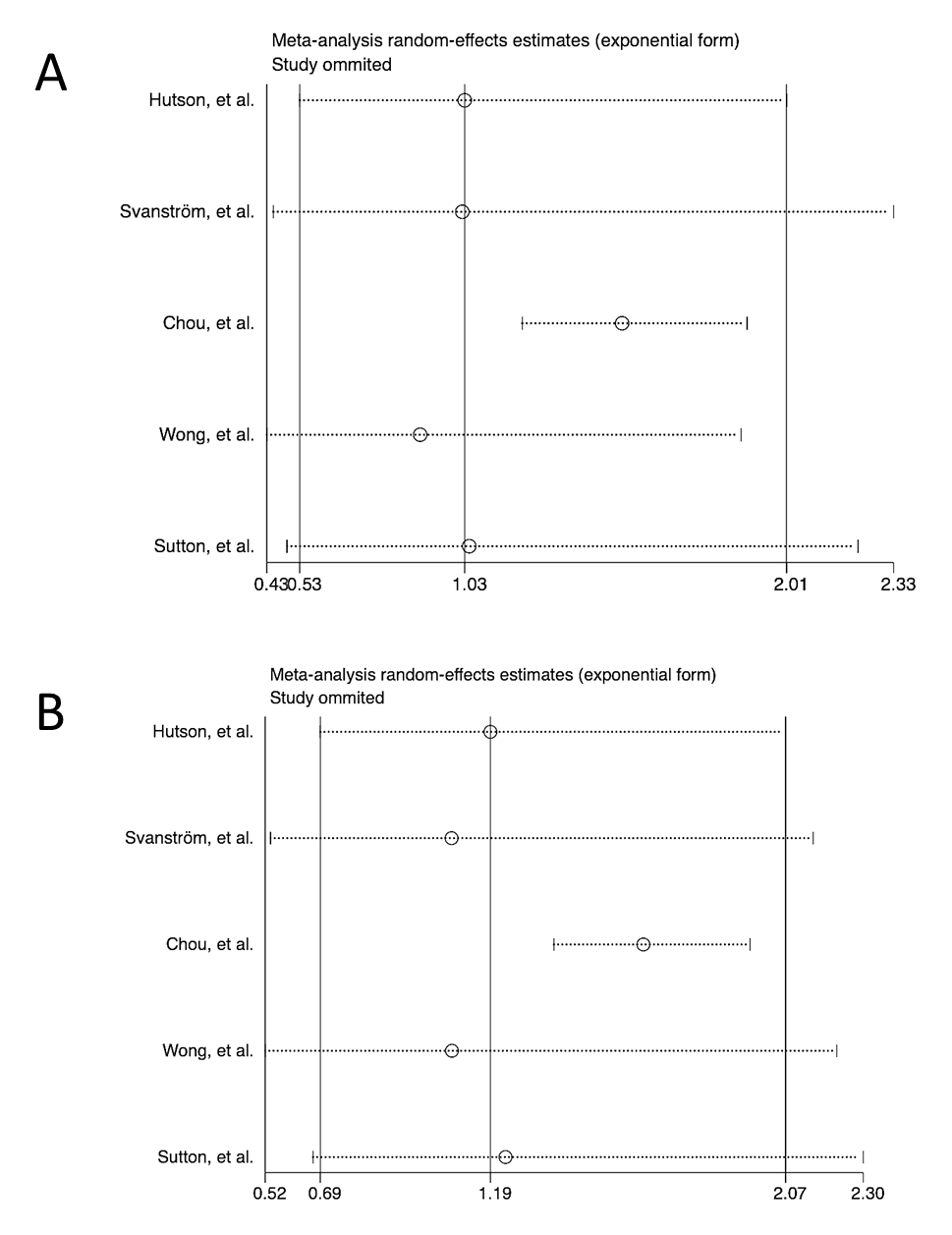

Supplement: S6 Fig — Leave-one-out analysis of observational studies with (A) short-term and (B) immediate outcomes of cardiac mortality showed no strong effects by any single study. (TIF) [file pone.0226637.s010.TIF]
